# Nanoantioxidants: Structure–Activity Relationships, Redox Mechanisms, Applications, and Safety

**DOI:** 10.3390/antiox15091177

**Published:** 2026-09-16

**Authors:** Min Ook Kim

**Affiliations:** Department of Civil Engineering, Seoul National University of Science and Technology, 232 Gongneung-ro, Nowon-gu, Seoul 01811, Republic of Korea; minookkim@seoultech.ac.kr

**Keywords:** nanoantioxidants, oxidative stress, nanomaterials, reactive oxygen species, nanozymes, redox regulation, nanotoxicology

## Abstract

Oxidative stress contributes to disease progression, food deterioration, agricultural losses, and material degradation, whereas many conventional antioxidants are limited by poor stability, low bioavailability, rapid consumption, or inadequate delivery. In this review, nanoantioxidants are defined as nanoscale systems intentionally designed to suppress or regulate damaging oxidative processes, and four functional categories are distinguished: intrinsically antioxidant nanomaterials, antioxidant nanozymes, antioxidant-functionalized nanoparticles, and nanocarriers used to deliver conventional antioxidants. The review systematically examines how particle size and surface area, morphology and exposed facets, oxidation state and defect density, surface chemistry and charge, aggregation, dissolution or ion release, dose, and the surrounding environment determine antioxidant activity. Particular emphasis is placed on the context-dependent transition from antioxidant to pro-oxidant behavior, the distinction between enzyme-like nanozyme activity and true enzymatic activity, and the need for nanoparticle-specific controls and complementary assays to avoid analytical interference. Evidence from chemical assays, cell culture, animal studies, and clinical research is considered separately because activity in a simplified chemical system does not by itself establish therapeutic efficacy or safety. Applications in nanomedicine, food preservation, agriculture, environmental protection, and engineering materials are critically compared. Long-term toxicity, biodistribution, clearance, degradation, environmental fate, and life-cycle impacts are highlighted as essential translational criteria; likewise, green synthesis is not treated as synonymous with sustainability without consideration of energy, solvents, purification, scalability, waste, and end-of-life behavior. Future progress will require standardized datasets and experimental protocols, mechanism-guided design, realistic validation, and carefully governed artificial-intelligence-assisted optimization.

## 1. Introduction

Reactive oxygen species (ROS) and reactive nitrogen species (RNS) are continuously generated through normal metabolic processes and environmental exposure. At physiological concentrations, these reactive species participate in cellular signaling, immune defense, and the regulation of proliferation and differentiation [1]. However, their excessive production or insufficient removal disrupts redox homeostasis and causes oxidative damage to lipids, proteins, nucleic acids, and cellular organelles [2]. Persistent oxidative stress is consequently associated with aging, inflammation, neurodegenerative and cardiovascular diseases, metabolic disorders, and cancer. Oxidative reactions also present technological challenges by accelerating food deterioration, reducing agricultural productivity, and shortening the functional life of susceptible materials [3,4]. Conventional antioxidants, including polyphenols, vitamins, enzymes, and synthetic radical scavengers, are widely employed to mitigate oxidative damage. Nevertheless, their effectiveness can be restricted by low aqueous solubility, chemical instability, poor bioavailability, rapid metabolism, and insufficient accumulation at target sites. These limitations are particularly relevant to poorly soluble bioactive compounds such as curcumin, for which nanoparticle encapsulation has been shown to improve oral bioavailability and pharmacokinetic performance [5]. Consequently, there is growing interest in antioxidant systems capable of providing improved stability, controlled release, sustained activity, and targeted delivery.

Within this review, “nanoantioxidants” is used as an umbrella term for nanoscale systems whose intended function is to reduce, intercept, or regulate damaging oxidative reactions. Four functional categories are distinguished. First, intrinsically antioxidant nanomaterials possess redox-active compositions or surfaces that directly scavenge radicals or decompose reactive oxygen species (ROS), as exemplified by functionalized fullerenes and several metal or metal-oxide nanomaterials [6,7,8,9,10,11]. Second, antioxidant nanozymes are a catalytic subset of nanomaterials that display superoxide dismutase (SOD)-, catalase (CAT)-, glutathione peroxidase (GPx)-, or related enzyme-like reactions under defined conditions [7,8,9,10,11,12]. Third, antioxidant-functionalized nanoparticles carry redox-active molecules or functional groups on their surfaces, so activity depends on ligand identity, loading, accessibility, and interfacial chemistry. Fourth, antioxidant nanocarriers are not necessarily intrinsically redox-active; instead, they improve the stability, solubility, targeting, pharmacokinetics, or controlled release of conventional antioxidant compounds [5]. Hybrid platforms can combine two or more of these functions.

This functional classification is complementary to the material-based classification used in Section 2. The distinction is important because an intrinsically catalytic nanoparticle, a nanozyme, a surface-functionalized particle, and an antioxidant-loaded carrier may all be described broadly as nanoantioxidants while exhibiting very different structure–activity relationships, assay requirements, safety profiles, and translational constraints [13,14,15,16,17,18,19]. Accordingly, the term is used here to describe intended redox function rather than to imply a single mechanism or an intrinsically beneficial biological effect.

Although nanoantioxidants are being explored in biomedical, food, agricultural, environmental, and engineering settings, the major unresolved challenge is reliable comparison across materials and experimental systems. Differences in synthesis, physicochemical characterization, dose expression, exposure conditions, biological models, and endpoint selection can produce apparently conflicting results. Conventional chemical antioxidant assays also may not adequately predict biological activity because nanoparticles can adsorb assay reagents, catalyze side reactions, scatter or absorb light, and quench or enhance fluorescence, thereby distorting measured antioxidant capacity [20]. Standardized characterization, orthogonal analytical methods, nanoparticle-only controls, and biologically relevant validation are therefore essential for establishing reproducible structure–activity relationships.

This article was developed as a structured narrative review rather than a systematic review or meta-analysis. The literature search was organized across Web of Science Core Collection, Scopus, PubMed, and Google Scholar using combinations of the terms nanoantioxidant, antioxidant nanoparticle, ROS-scavenging nanomaterial, nanozyme, cerium oxide, manganese oxide, polydopamine, carbon dot, antioxidant nanocarrier, pro-oxidant, oxidative stress, SOD-like, catalase-like, GPx-like, safety, toxicity, biodistribution, green synthesis, sustainability, and machine learning. Peer-reviewed primary studies and reviews were included when they provided mechanistic, structure–activity, assay, application, safety, or sustainability information directly relevant to nanoantioxidants. Studies focused exclusively on non-nanoscale antioxidants, or on intentionally ROS-generating nanomaterials without relevance to antioxidant/pro-oxidant duality, were excluded from the core synthesis. Duplicate records were screened by title, DOI, and bibliographic information, and reference lists of key publications were examined to identify additional relevant studies. Because the objective was critical integration across biomedical, food, agricultural, environmental, and engineering disciplines rather than quantitative evidence pooling, no meta-analysis was performed.

**Figure 1:**
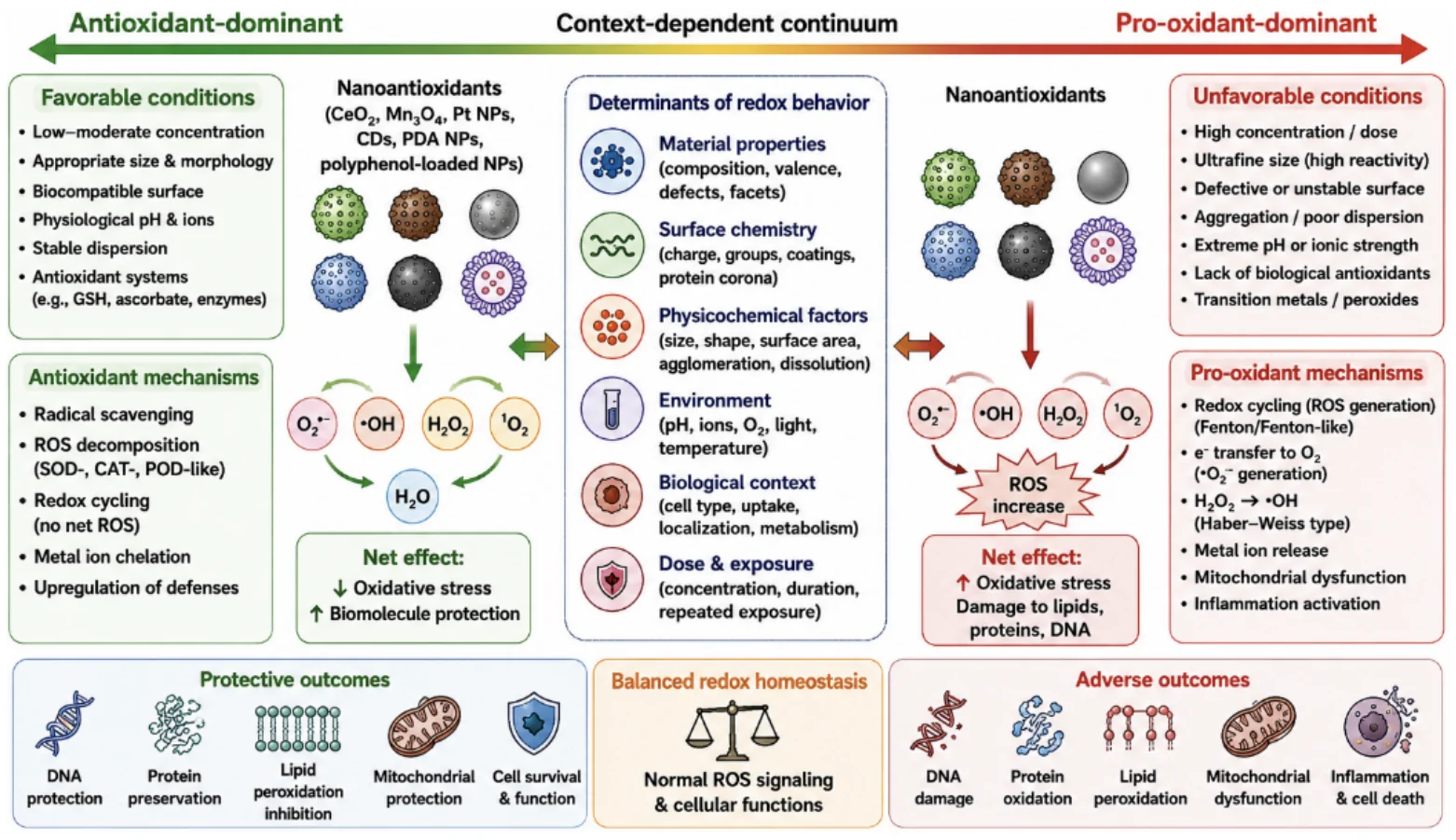
Context-dependent redox behavior of nanoantioxidants [1,2,4,7,8,9].

This review critically examines the principal functional and material classes of nanoantioxidants and evaluates how their physicochemical characteristics govern redox activity. Particular attention is given to structure–activity relationships, direct radical scavenging, enzyme-like catalysis, metal-ion regulation, reactive-species decomposition, and antioxidant delivery. The review also distinguishes chemical antioxidant activity from evidence obtained in cell culture, animal models, and clinical research; evaluates assay interference and appropriate controls; and compares application-specific benefits and limitations across biomedical, food, agricultural, environmental, and engineering settings. Finally, biocompatibility, long-term safety, biodistribution and clearance, environmental sustainability, standardization, clinical translation, and opportunities for artificial-intelligence-assisted and safe-by-design development are considered. By connecting nanoscale structure with mechanism, evidence level, application, and safety, the review aims to provide a framework for designing nanoantioxidants that are effective under defined conditions rather than merely highly active in simplified screening assays Figure 1 illustrates this context-dependent redox continuum [1,2,4,7,8,9].

## 2. Classification and Design of Nanoantioxidants

Nanoantioxidants can be organized using both functional and compositional criteria. Functionally, two related but distinct concepts should be separated: intrinsic nanoantioxidants, in which the nanomaterial itself participates in redox reactions, and antioxidant nanocarriers, in which the nanoscale platform primarily protects and delivers a molecular antioxidant. Hybrid systems may combine intrinsic redox activity with cargo delivery. This functional distinction is important because catalytic activity, dose normalization, biological interaction, and safety assessment differ substantially between a redox-active nanoparticle and a carrier whose antioxidant effect originates mainly from its payload.

Nanoantioxidants can be categorized in two complementary ways. Material-based classes include metal- and metal-oxide-based, carbon-based, polymeric and lipid-based, and biogenic or hybrid nanomaterials, whereas functional classes distinguish intrinsically antioxidant materials, catalytic nanozymes, antioxidant-functionalized nanoparticles, and antioxidant-delivery nanocarriers. These categories should not be conflated: for example, a polymeric nanoparticle may be an essentially inert carrier, a redox-active polymeric antioxidant, or a hybrid system containing a catalytic inorganic component. Intrinsic and nanozyme systems are governed primarily by exposed active sites, oxidation states, defects, and interfacial chemistry; antioxidant-functionalized nanoparticles depend strongly on the density and accessibility of surface-bound antioxidant groups; and nanocarriers depend mainly on cargo loading, protection, release kinetics, and biological or environmental transport. Table 1 therefore compares material platforms while the accompanying discussion emphasizes these mechanistic distinctions, major advantages, and limitations.

**Figure 2:**
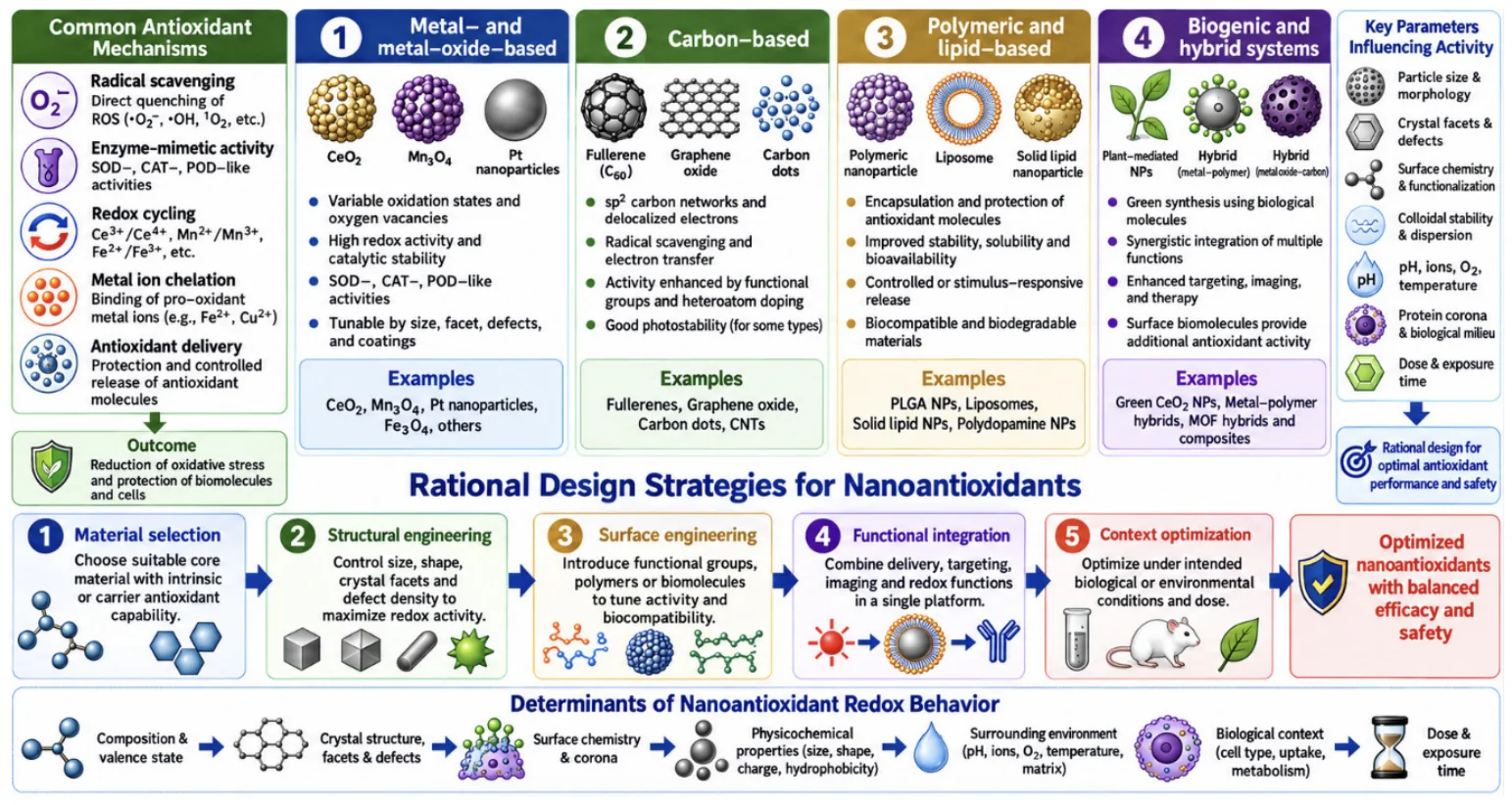
Classification and design of nanoantioxidants [7,8,21].

As shown in Figure 2, four broad compositional platforms can be considered, while the functional distinction between intrinsically active nanoantioxidants and antioxidant nanocarriers provides a second, complementary level of classification. This hierarchical approach avoids treating CeO_2_-, Mn-, and noble-metal systems as separate classes and emphasizes that composition alone does not determine antioxidant function [7,8,21,22,23,24,25,26,27,28,29,30,31].

### 2.1. Metal- and Metal-Oxide-Based Nanoantioxidants

Metal and metal-oxide nanoparticles are among the most extensively investigated intrinsic nanoantioxidants because variable oxidation states and defect structures can support catalytic redox reactions. Nanoceria provides a useful structure–activity example: the surface Ce^3+^/Ce^4+^ ratio is coupled to oxygen-vacancy density and electron-transfer capacity, so changes in particle size, exposed facets, surface Ce^3+^ concentration, ligands, or surrounding ions can alter SOD- and CAT-like activity [7,8,9,10,11,13,16]. Increasing accessible defect-rich surface sites may enhance particular redox pathways, whereas surface passivation or environment-induced valence shifts can attenuate or redirect activity. Thus, nominal composition alone is insufficient to predict nanoceria behavior; mechanism-guided design requires control of the surface oxidation state and vacancy environment under the intended exposure conditions.

Other transition-metal oxides exhibit related enzyme-mimetic behavior. Manganese oxide nanomaterials, for example, can participate in redox reactions involving superoxide, hydrogen peroxide, and other reactive intermediates. Mn_3_O_4_ nanoparticles have demonstrated multienzyme-like antioxidant properties, illustrating how mixed oxidation states and surface redox centers can generate broad ROS-scavenging activity [12]. Such nanozyme behavior has expanded the concept of antioxidant nanomaterials from passive radical scavengers toward catalytic systems capable of continuously regulating oxidative environments.

Noble-metal nanoparticles can also display pronounced antioxidant activity. Platinum nanoparticles are particularly notable because their surfaces can catalyze reactions involving superoxide and hydrogen peroxide and thereby mimic multiple endogenous antioxidant enzymes [21]. Their high catalytic stability makes them attractive for persistent ROS regulation. Nevertheless, antioxidant activity depends strongly on particle size, surface ligands, aggregation state, dose, and surrounding chemical conditions. Metallic nanoparticles may under some circumstances catalyze ROS production rather than elimination, demonstrating that nanoscale redox activity is intrinsically context dependent [22].

Accordingly, rational design of metal-based nanoantioxidants requires control of oxidation state, exposed crystal facets, defect chemistry, surface coatings, and dissolution behavior. Surface functionalization may improve colloidal stability and biocompatibility while simultaneously modifying access of ROS to catalytic sites. Optimization should therefore focus not simply on maximizing catalytic activity but on maintaining a favorable antioxidant/pro-oxidant balance within the intended exposure environment.

### 2.2. Carbon-Based Nanoantioxidants

Carbon-based nanomaterials constitute another major nanoantioxidant platform and include fullerenes, carbon nanotubes, graphene derivatives, carbon dots, and related nanostructures. Their antioxidant properties originate from delocalized π-electron systems, electron-transfer capability, surface functional groups, and, in some cases, direct addition reactions with radical species. Fullerenes are among the earliest carbon nanomaterials recognized for radical-scavenging behavior because their conjugated carbon framework can accommodate multiple radical species.

Chemical functionalization is particularly important because pristine carbon nanostructures often exhibit limited aqueous dispersibility and may interact nonspecifically with biological components. Introduction of hydroxyl, carboxyl, amino, or polymeric surface groups can improve dispersion and alter electronic characteristics, thereby modifying antioxidant activity and biological compatibility. Functionalized fullerene derivatives, graphene-based materials, and carbon dots have consequently been investigated as tunable radical-scavenging systems [23,24].

Carbon dots are particularly attractive because their small dimensions, abundant surface functionalities, relatively straightforward synthesis, and optical properties enable antioxidant functionality to be combined with imaging or sensing. Their redox behavior can be adjusted through precursor selection, heteroatom doping, surface passivation, and functionalization. These characteristics provide opportunities for multifunctional nanoantioxidants in which ROS modulation is integrated with diagnostic or therapeutic functions. However, carbon nanomaterials cannot universally be regarded as antioxidants. Their biological effects depend on structural defects, residual catalysts, surface chemistry, aggregation, concentration, and irradiation conditions. Certain graphene derivatives and carbon nanotubes, for example, may induce oxidative stress under unfavorable conditions. The design of carbon-based nanoantioxidants must therefore account for both radical-scavenging capacity and potential ROS-generating pathways.

### 2.3. Polymeric and Lipid-Based Nanoantioxidants

Polymeric and lipid-based nanosystems should be distinguished from intrinsically redox-active nanoantioxidants when their principal function is delivery. In these antioxidant nanocarriers, the carrier may have little or no intrinsic catalytic antioxidant activity; instead, nanoscale formulation improves the solubility, chemical stability, protection, transport, localization, and release of molecular antioxidants. Their efficacy should therefore be evaluated using loading, release kinetics, bioaccessibility or pharmacokinetics, and payload-specific antioxidant outcomes rather than being compared directly with the catalytic activity of inorganic nanozymes.

Polymeric nanoparticles fabricated from biodegradable materials such as poly(lactic-co-glycolic acid) (PLGA), chitosan, and related polymers have been widely explored as carriers for polyphenols and other natural antioxidants. Encapsulation of curcumin, resveratrol, quercetin, and related molecules can mitigate limitations associated with poor aqueous solubility, rapid degradation, and limited bioavailability [5,25]. Nanocarriers may also enable controlled or stimulus-responsive release, potentially concentrating antioxidant activity at sites characterized by elevated oxidative stress.

Lipid-based systems—including liposomes, solid lipid nanoparticles, and nanostructured lipid carriers—offer complementary advantages, particularly for lipophilic antioxidants. Their amphiphilic organization allows incorporation of compounds with different polarity while protecting oxidation-sensitive cargo from unfavorable external conditions. Liposomal and lipid-nanoparticle formulations can consequently enhance antioxidant stability and facilitate delivery across biological interfaces [26].

An increasingly important development is the emergence of polymers that themselves possess redox-active functionality. Polydopamine nanoparticles provide a prominent example because catechol and quinone groups within their structure can participate directly in radical scavenging and redox reactions [27,28]. Such systems blur the distinction between antioxidant carrier and antioxidant active material, allowing structural, delivery, adhesive, photothermal, and redox functions to be incorporated within a single nanoscale platform.

### 2.4. Biogenic and Hybrid Nanoantioxidants

Biogenic synthesis provides an alternative strategy for producing nanoantioxidants using plant extracts, microorganisms, proteins, polysaccharides, or other biological components as reducing, stabilizing, or functionalizing agents. Phytochemicals such as polyphenols and flavonoids can simultaneously facilitate nanoparticle formation and remain associated with nanoparticle surfaces, potentially contributing additional antioxidant functionality [29].

Plant-mediated metallic and metal-oxide nanoparticles have received considerable attention as potentially lower-hazard alternatives to some conventional synthesis routes. Their measured antioxidant behavior may arise from the inorganic nanoparticle core, residual plant metabolites, deliberately retained surface-capping molecules, or a combination of these contributions [29,30]. Variation in extract composition, growth conditions, synthesis parameters, and purification can therefore produce substantial batch-to-batch differences, making standardization and rigorous physicochemical characterization essential before different biogenic preparations are compared.

Attribution of antioxidant activity in biogenic nanoparticles requires explicit controls. Ideally, antioxidant measurements should compare the precursor extract, reaction supernatant, thoroughly washed nanoparticle fraction, and, where feasible, desorbed or quantified surface-bound organics. A reduction in apparent activity after repeated purification would indicate that residual phytochemicals contribute substantially to the measured response, whereas persistent activity in well-purified particles would support a stronger contribution from the nanoparticle core or stable surface chemistry. Without such controls, strong results from DPPH-, ABTS-, or related chemical assays should not automatically be assigned to the nanoparticle itself.

Hybrid nanoantioxidants intentionally combine different material classes to exploit complementary properties. Examples include metal–polymer, metal oxide–carbon, lipid–polymer, and biomolecule–inorganic nanostructures. Hybridization can integrate catalytic ROS decomposition, radical scavenging, targeted delivery, imaging, and stimulus-responsive release within a single system. Biomimetic approaches, including protein-, membrane-, and enzyme-associated nanostructures, further seek to reproduce or reinforce endogenous antioxidant defense pathways [31].

The principal advantage of hybrid design is functional integration; however, increasing structural complexity may also complicate reproducibility, characterization, biodegradation, safety evaluation, and manufacturing. Thus, multifunctionality should be balanced against translational simplicity.

### 2.5. Cross-Cutting Principles for Rational Design

Across nanoantioxidant classes, rational design should begin with a defined set of physicochemical descriptors that can be linked quantitatively to function. Particle size and specific surface area influence the number of accessible reactive sites and transport behavior; morphology and crystal facets determine the exposure of catalytic centers; oxidation state and defect density regulate electron transfer; and surface chemistry controls dispersion, interfacial reactions, and biological recognition [31,32,33]. These descriptors should be measured in the formulation actually used for testing rather than inferred solely from the as-synthesized material.

The effective biological identity of a nanoantioxidant can differ substantially from its initial synthetic identity. Aggregation, dissolution, oxidation or reduction, ligand exchange, adsorption of proteins and other biomolecules, and formation of a biomolecular corona can change size, surface charge, catalytic accessibility, cellular uptake, and clearance [10,14,15]. Consequently, activity measured in a simplified chemical buffer should not be extrapolated directly to cells or organisms without confirming how the nanoparticle transforms in the relevant medium and what material state is actually encountered by biological systems.

Practical design criteria should therefore extend beyond a high value in a single antioxidant assay. A candidate nanoantioxidant should have well-defined and reproducible physicochemical properties, a mechanism-based activity profile, appropriate colloidal or formulation stability, biological compatibility, controlled persistence or degradability, and a dose window that remains effective without inducing pro-oxidant effects. Standardized testing and transparent reporting are essential so that changes in activity can be attributed to material design rather than batch variability or assay conditions [13,14,15,16,17,20,32,33].

Environmental performance is an additional design constraint. Biodegradability, persistence, dissolution and metal-ion release, transformation in water or soil, and the potential for accumulation or non-target ecological effects should be considered alongside antioxidant efficacy, particularly for materials intended for repeated or large-scale use. These criteria provide a bridge between structure–activity optimization and the safety and life-cycle considerations discussed in later sections.

Accordingly, the optimal nanoantioxidant is not necessarily the material with the greatest radical-scavenging capacity under idealized conditions. It is a reproducible system in which composition, structure, surface chemistry, catalytic or delivery mechanism, exposure behavior, safety, and persistence are controlled for a defined application. This mechanism-guided and application-specific framework provides the basis for interpreting the antioxidant pathways discussed in Section 3 [20,31,32,33].

## 3. Antioxidant Mechanisms of Nanomaterials

**Figure 3:**
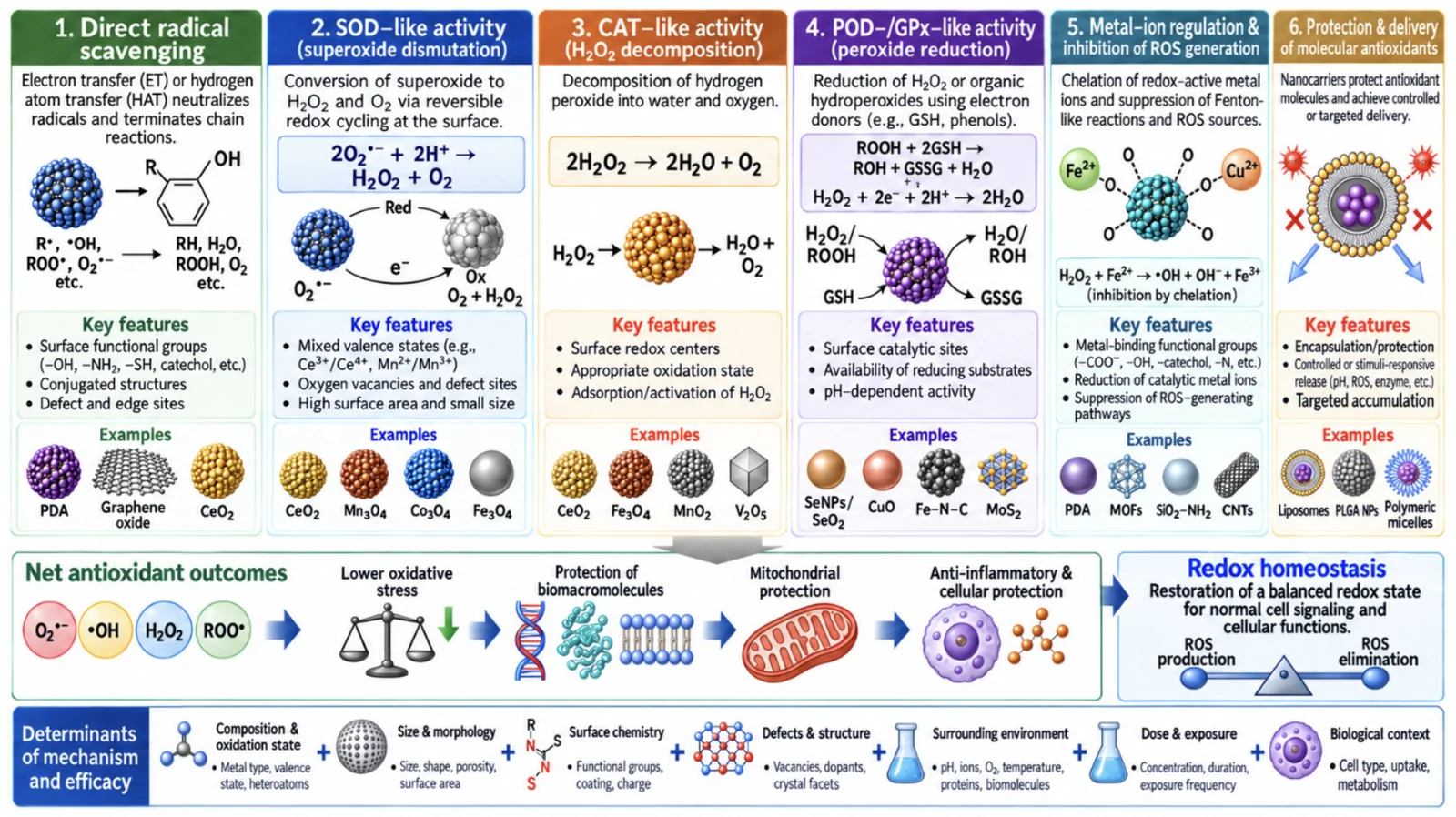
Major antioxidant mechanisms and enzyme-like pathways of nanoantioxidants [32,33,34].

Section 2 addressed how material structure influences redox behavior; the present section focuses on the chemical and biological mechanisms through which that behavior is expressed. Nanoantioxidants may directly neutralize radicals, catalytically convert reactive species, regulate redox-active metal ions, modulate endogenous antioxidant pathways, or protect and deliver molecular antioxidants [32,33,34,35,36,37,38]. Importantly, enzyme-mimetic labels describe catalytic functions rather than the net biological outcome: SOD-like, CAT-like, GPx-like, or peroxidase (POD)-like activity does not by itself prove that a nanomaterial is an antioxidant. The overall effect depends on the complete reaction network, substrate availability, competing reactions, and the balance between species removed and species generated. This distinction is especially important for POD-like and Fenton/Fenton-like pathways, which can increase hydroxyl-radical production under some conditions. Table 2 summarizes the major mechanistic functions and their limitations, and Figure 3 illustrates how these pathways can contribute to integrated redox regulation.

### 3.1. Direct Radical Scavenging and Electron/Hydrogen Transfer

Direct radical scavenging represents one of the most fundamental antioxidant mechanisms. Reactive species such as superoxide (O_2_•^−^), hydroxyl radicals (•OH), peroxyl radicals (ROO•), and other radical intermediates can be neutralized through electron transfer (ET), hydrogen atom transfer (HAT), proton-coupled electron transfer, or radical addition reactions at the nanoparticle surface. These processes interrupt radical-chain reactions and thereby limit oxidative damage to lipids, proteins, nucleic acids, and other susceptible molecules [32,35].

Carbonaceous and polymeric nanoantioxidants are particularly relevant to this mechanism. Their conjugated structures and surface functional groups can stabilize unpaired electrons and facilitate electron donation or acceptance. In polydopamine (PDA), for example, catechol/quinone redox couples and phenolic hydroxyl groups enable H-atom and electron transfer, allowing PDA-based nanostructures to neutralize several ROS and radical intermediates [35,36]. The reversible nature of these redox-active groups may provide more sustained activity than a simple one-time radical-quenching event.

Direct scavenging is nevertheless highly surface dependent. Increasing specific surface area can expose additional redox-active sites, while functionalization can alter electron density, accessibility, hydrophilicity, and interaction with reactive species. Conversely, aggregation or excessive surface passivation may decrease the number of accessible active sites. Thus, the radical-scavenging efficiency of a nanoantioxidant reflects both its intrinsic chemical structure and the accessibility of reactive groups at the nano–environment interface.

Reactive nitrogen species (RNS) should be considered alongside ROS when evaluating redox regulation. Peroxynitrite (ONOO−), formed by the rapid reaction of superoxide with nitric oxide, can oxidize and nitrate biomolecules and is therefore a relevant target in oxidative/nitrosative stress [2]. Nanoantioxidants may influence RNS either directly through surface electron-transfer or radical-scavenging reactions or indirectly by lowering superoxide availability and thereby limiting ONOO− formation. However, RNS interactions are less consistently characterized than ROS pathways, so claims of broad “redox scavenging” should distinguish ROS-specific measurements from direct evidence for RNS modulation [2,32].

### 3.2. Superoxide Dismutase-like Activity

SOD constitutes a major component of endogenous antioxidant defense by catalyzing the disproportionation of superoxide into molecular oxygen and hydrogen peroxide:

(1) $$2O_{2}^{•-}+2H^{+}\to H_{2}O_{2}+O_{2}$$

In metal-oxide nanozymes, surface redox centers can serve as electron donors and acceptors during superoxide conversion. Cerium-, manganese-, and other transition-metal-containing nanostructures can therefore exhibit SOD-like catalytic activity when suitable oxidation states coexist at their surfaces [32,33]. For mechanistic interpretation, the key question is not simply whether SOD-like activity is detected, but whether the measured rate, substrate specificity, and downstream peroxide handling produce a favorable net redox effect under the relevant conditions.

SOD-like activity should consequently be quantified with kinetic or ROS-specific measurements and interpreted separately from cellular protection. Changes in size, defect accessibility, ligand coverage, or aggregation may modify catalytic rates, but these structure-dependent effects should be linked to measured reaction parameters rather than assumed from particle dimensions alone.

### 3.3. Catalase-like Decomposition of Hydrogen Peroxide

Although SOD-like conversion removes superoxide, it generates H_2_O_2_, which remains biologically reactive and can generate highly damaging •OH in the presence of suitable redox-active metals. Effective antioxidant systems therefore frequently require complementary CAT-like activity. Catalase converts hydrogen peroxide into water and molecular oxygen:

(2) $$2H_{2}O_{2}\to 2H_{2}O+O_{2}$$

Multienzyme-mimetic nanoantioxidants capable of combining SOD- and CAT-like activities are particularly attractive because they can sequentially remove $O_{2}^{•-}$ and its H_2_O_2_ product. Such cascade catalysis provides a more complete antioxidant pathway than selective removal of only one ROS [33,34].

CAT-like behavior is commonly mediated by reversible electron-transfer reactions at metal-containing catalytic sites. The activity is sensitive to surface oxidation state, local coordination, pH, substrate concentration, and adsorption of H_2_O_2_ to catalytic centers. These factors can alter not only reaction rates but also reaction pathways. Consequently, a nanomaterial exhibiting efficient H_2_O_2_ decomposition under one set of conditions may display substantially different behavior in another chemical or biological microenvironment [32,33,34]. This environmental sensitivity has important implications for nanoantioxidant design. Catalytic activity should therefore be evaluated under physiologically or technologically relevant conditions rather than inferred exclusively from simplified chemical assays.

### 3.4. Peroxidase- and Glutathione Peroxidase-like Pathways

POD-like activity requires particularly cautious interpretation. Natural peroxidases couple peroxide reduction to defined electron-donor substrates, whereas metal-containing nanomaterials can catalyze several peroxide-dependent pathways whose products vary with pH, oxidation state, coordination environment, substrate availability, and H_2_O_2_ concentration [32,34]. Accordingly, observation of a POD-like rate in a model chromogenic assay does not by itself demonstrate antioxidant function. Some materials instead favor Fenton or Fenton-like conversion of H_2_O_2_ to highly reactive •OH and therefore operate as pro-oxidants. The relevant criterion is the net change in individual ROS and oxidative damage across the complete catalytic cycle, not the assignment of an enzyme-like label.

GPx-like nanozymes provide another route for peroxide removal. The designation “GPx-like” should likewise be reserved for systems in which peroxide reduction is demonstrated together with an appropriate reducing co-substrate or thiol-coupled reaction, rather than inferred from nonspecific H_2_O_2_ loss. Selenium-, tellurium-, metal-, and carbon-containing nanostructures have been explored as GPx mimics because suitable catalytic centers can facilitate thiol-mediated reduction of H_2_O_2_ or organic hydroperoxides [32,37]. Kinetic analysis, reaction-product identification, and controls for spontaneous peroxide decomposition are important for distinguishing genuine catalytic mimicry from adsorption, probe interference, or parallel redox reactions.

The development of nanoantioxidants possessing combined SOD-, CAT-, and GPx-like functions is therefore particularly promising. Such systems more closely reproduce the cooperative nature of endogenous antioxidant networks and may provide broader protection against heterogeneous ROS.

### 3.5. Metal-Ion Regulation and Inhibition of ROS Generation

Nanoantioxidants can also reduce oxidative stress indirectly by regulating redox-active metal ions or suppressing ROS-generating reactions. Free Fe^2+^ and Cu^+^, for example, can participate in reactions that convert relatively moderate oxidants such as H_2_O_2_ into highly reactive •OH. Materials containing metal-binding functional groups can decrease the availability of these catalytic ions and thereby suppress radical formation.

Surface hydroxyl, catechol, carboxyl, amino, and other coordinating groups may contribute to metal-ion binding. Carbonaceous, polymeric, and biomolecule-functionalized nanoparticles can therefore combine direct radical scavenging with metal-ion regulation. In PDA-based systems, catechol groups are particularly relevant because they participate both in redox reactions and metal coordination [35,36].

Nanoantioxidants may also regulate oxidative stress indirectly by influencing endogenous cellular defense systems. In addition to altering mitochondrial ROS generation and inflammatory oxidant production, some nanomaterial interventions can affect nuclear factor erythroid 2-related factor 2 (Nrf2)/antioxidant response element (ARE) signaling, glutathione synthesis and redox cycling, and the expression or activity of endogenous superoxide dismutase, catalase, and glutathione peroxidase. Such effects can amplify or prolong antioxidant protection without requiring continuous direct scavenging of every reactive species. However, pathway activation should be demonstrated using appropriate molecular or biochemical endpoints because an observed decrease in cellular ROS does not, by itself, identify the responsible mechanism [32,36].

### 3.6. Protection and Delivery of Molecular Antioxidants

A mechanistically distinct antioxidant strategy involves using nanomaterials as carriers rather than relying solely on intrinsic nanoparticle redox activity. Conventional antioxidants such as polyphenols often suffer from poor aqueous solubility, chemical instability, rapid metabolism, and limited accumulation at sites of oxidative stress. Nanocarriers can protect these molecules from premature degradation and provide controlled or targeted delivery [25,38].

Polymeric nanoparticles, lipid nanoparticles, liposomes, and hybrid nanostructures can encapsulate antioxidant molecules and modify their dissolution, transport, cellular uptake, and release profiles. Stimuli-responsive platforms may further release antioxidant cargo in response to ROS, pH, enzymes, or other characteristics of pathological microenvironments. This strategy can increase local antioxidant concentrations while reducing unnecessary systemic exposure.

More sophisticated platforms combine carrier-mediated delivery with intrinsic antioxidant activity. For example, a redox-active nanoparticle may directly scavenge ROS while simultaneously delivering a molecular antioxidant, enzyme, anti-inflammatory compound, or other therapeutic cargo. Such cooperative mechanisms may produce greater protection than either component alone, although mechanistic synergy should be demonstrated experimentally rather than inferred from formulation complexity.

### 3.7. From Individual Mechanisms to Integrated Redox Regulation

The mechanisms described above rarely operate independently. A single nanoantioxidant may simultaneously exhibit direct radical scavenging, SOD-like activity, CAT-like activity, metal-ion binding, antioxidant delivery, and biological redox regulation. The dominant pathway depends on material composition and structure as well as the surrounding chemical and biological context [32,33,34].

This mechanistic multiplicity represents both an advantage and a validation challenge. Chemical radical-scavenging capacity, catalytic ROS decomposition, and cellular antioxidant effects are distinct endpoints and should not be treated as interchangeable. Mechanistic validation should combine ROS- or RNS-specific measurements with appropriate kinetic parameters, oxidation-state and surface characterization before and after reaction, nanoparticle-only and assay-interference controls, and biologically relevant models. Where enzyme-mimetic activity is claimed, substrate dependence and reaction products should be verified; where cellular protection is claimed, the contribution of uptake, localization, endogenous defense pathways, and possible assay artifacts should be separated. Such orthogonal evidence is necessary to determine whether an apparent antioxidant effect reflects true reactive-species removal, redistribution between species, altered production, or measurement interference [20,32,33,34,35,36,37,38].

Ultimately, the relevant endpoint is the net effect on redox homeostasis rather than the magnitude of any single antioxidant reaction. An effective nanoantioxidant should selectively attenuate pathological oxidative or nitrosative stress while preserving physiological ROS–RNS signaling required for cellular communication, host defense, and homeostasis. It should also maintain its intended mechanism in the target environment without introducing secondary pro-oxidant pathways. This principle provides the mechanistic bridge from catalytic measurements to the biological, application, and safety considerations discussed in the following sections.

### 3.8. Antioxidant–Pro-Oxidant Balance

Antioxidant and pro-oxidant behavior should be treated as two possible outcomes of the same nanoscale redox system rather than as fixed material identities. Dose and exposure duration are central determinants: increasing concentration can improve ROS-scavenging capacity up to an optimum but can also increase particle–cell contact, catalytic turnover, local substrate depletion, membrane interactions, or metal-ion release. A protective concentration window established in one assay therefore cannot be assumed to apply to another biological or technological environment [13,14,15,16,17].

pH, oxidation state, dissolution, and ion release can change both reaction rates and reaction direction. Acidic compartments may accelerate dissolution of some metal-containing nanoparticles, while changes in surface valence state or ligand coordination can alter electron-transfer pathways. Released Fe, Cu, or other redox-active ions may promote Fenton/Fenton-like ROS generation; conversely, favorable valence-state cycling in nanoceria or manganese oxides can support catalytic ROS removal. Thus, the same nominal composition can display different net redox behavior depending on local pH, oxygen, H_2_O_2_ concentration, phosphate, reductants, and competing ligands.

Surface chemistry and biological context further modify this balance through aggregation, protein-corona formation, membrane binding, subcellular localization, and interactions with endogenous antioxidants and enzymes. Evaluation of redox behavior should therefore report the physicochemical state of the material during exposure and should quantify both ROS removal and possible ROS generation. Where possible, antioxidant efficacy should be interpreted together with oxidative-damage endpoints, cell viability or tissue injury, and changes in endogenous redox signaling rather than from a single radical-scavenging value.

### 3.9. Antioxidant Activity Assays, Nanoparticle Controls, and Evidence Interpretation

Chemical antioxidant assays are useful for screening but are especially vulnerable to nanoparticle-specific artifacts. Nanoparticles can absorb or scatter the assay wavelength, quench or enhance fluorescence, adsorb dyes or radicals, catalyze probe conversion, alter pH, or continue reacting during optical readout [20]. Appropriate controls should therefore include nanoparticle-only blanks at each tested concentration, reagent-only controls, time-matched optical backgrounds, and, when relevant, controls for dissolved ions, synthesis supernatants, capping agents, or antioxidant cargo. For strongly absorbing or scattering particles, separation of particles from the reaction mixture before optical measurement should be considered only when it does not remove the analyte or otherwise alter the reaction endpoint.

No single assay is sufficient to establish antioxidant performance. Results from colorimetric or fluorescence assays should be confirmed with complementary methods based on different detection principles, such as radical-specific electron paramagnetic resonance/spin trapping, direct peroxide or superoxide measurements, chromatographic or mass-spectrometric product analysis, catalytic kinetic measurements, or biologically relevant oxidative-damage markers. Agreement among orthogonal methods is particularly important when a nanomaterial has strong optical, adsorptive, catalytic, or magnetic properties that overlap with the assay readout.

Evidence should also be interpreted hierarchically. A positive result in DPPH, ABTS, FRAP, ORAC, or another chemical assay demonstrates reactivity under that assay condition but does not establish cellular protection, bioavailability, therapeutic efficacy, or safety. Cell-culture studies add information on uptake, intracellular ROS, cytotoxicity, and signaling but do not reproduce whole-body pharmacokinetics or long-term organ exposure. Animal studies can address biodistribution, efficacy, clearance, and systemic toxicity, whereas clinical evidence is required before therapeutic benefit in humans can be claimed. Accordingly, conclusions in this review distinguish chemical activity, in vitro biological effects, in vivo preclinical evidence, and clinical evidence whenever the underlying literature permits.

## 4. Biomedical and Healthcare Applications of Nanoantioxidants

**Figure 4:**
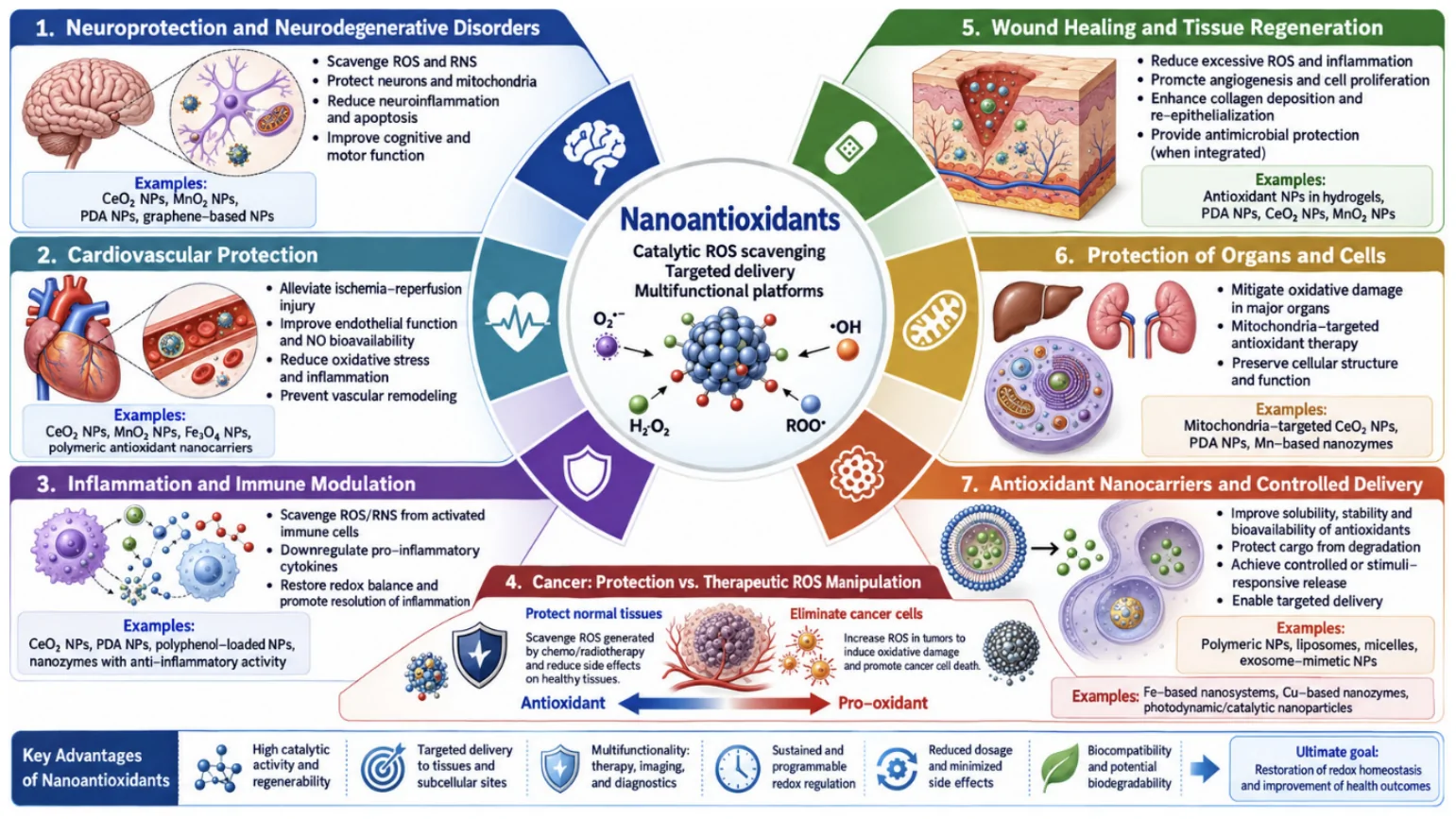
Biomedical and healthcare applications of nanoantioxidants [39,40,41].

Biomedical nanoantioxidant research spans a wide evidentiary spectrum, from chemical and cell-based studies to animal models, while clinical evidence remains limited for most intrinsically redox-active nanozymes. Accordingly, application claims should distinguish proof-of-mechanism from demonstrated therapeutic benefit. The systems discussed below include catalytic nanoparticles, antioxidant nanocarriers, and multifunctional platforms investigated for neuroprotection, cardiovascular injury, inflammation, cancer-related redox control, wound healing, and tissue protection [39,40,41,42,43,44,45,46,47,48,49,50,51,52,53]. Across these applications, efficacy depends not only on antioxidant potency but also on dose, tissue exposure, cellular uptake, subcellular localization, pharmacokinetics, and clearance. A common design objective is selective suppression of pathological oxidative stress without indiscriminate removal of physiological redox signals. Major biomedical applications are summarized in Figure 4 below.

### 4.1. Neuroprotection and Neurodegenerative Disorders

The nervous system is particularly susceptible to oxidative injury because of its high oxygen consumption, oxidation-sensitive lipids, and dependence on mitochondrial function. Evidence for neuroprotective nanoantioxidants is predominantly preclinical, including cellular and animal studies of ceria- and other nanozyme-based systems in models of neurodegeneration and ischemic injury [39,42,44,50]. These studies support biological plausibility but do not by themselves establish clinical efficacy.

Delivery to the central nervous system is a major practical constraint. Blood–brain barrier transport, protein-corona formation, systemic biodistribution, cellular uptake, intracellular trafficking, metabolism or transformation, and renal, hepatic, or reticuloendothelial clearance can substantially change the exposure achieved at neural targets. Surface functionalization may improve transport or cell-specific uptake, but it can also alter immune recognition and long-term retention. Neuroprotective nanoantioxidants should therefore be evaluated using quantitative biodistribution and clearance data alongside redox endpoints, rather than treating delivery and safety as secondary considerations.

### 4.2. Cardiovascular Protection

Oxidative stress contributes to endothelial dysfunction, ischemia–reperfusion injury, vascular inflammation, and pathological remodeling. Nanozyme-enabled cardiovascular protection has shown encouraging results mainly in experimental and preclinical models, where catalytic ROS regulation or targeted delivery can reduce oxidative injury [43,44]. Translation will require confirmation that the effective tissue concentration can be achieved without disturbing vascular redox signaling or producing long-term nanoparticle accumulation.

ROS-scavenging nanoparticles are particularly attractive for ischemia–reperfusion conditions, in which rapid ROS generation occurs following restoration of oxygen supply. Nanomaterials possessing SOD- and CAT-like activities may sequentially convert superoxide and hydrogen peroxide into less reactive products, reducing oxidative injury during reperfusion. Antioxidant polymeric nanoparticles and redox-responsive nanocarriers can additionally be designed to accumulate in inflamed or damaged vascular tissues and release therapeutic cargo in response to local oxidative conditions. Across cardiovascular applications, dose and targeting should therefore be optimized to restore redox balance rather than to eliminate ROS indiscriminately.

### 4.3. Inflammation and Immune Modulation

Oxidative stress and inflammation are closely interconnected. Activated immune cells generate ROS and reactive nitrogen species as part of host defense, whereas excessive ROS can activate redox-sensitive inflammatory pathways and promote further cytokine production. This reciprocal amplification contributes to persistent inflammatory microenvironments.

Nanoantioxidants can interrupt this cycle by reducing excessive ROS while modulating downstream inflammatory responses. Catalytic nanoparticles, PDA-based systems, and other ROS-responsive nanomaterials have demonstrated potential to decrease oxidative stress and inflammatory signaling simultaneously [40,45]. Such dual activity is particularly relevant to chronic inflammatory diseases, in which antioxidant treatment alone may be insufficient if inflammatory ROS production remains continuously activated.

The biological outcome, however, depends strongly on nanoparticle–immune system interactions. Surface charge, size, shape, coating, aggregation state, and protein-corona formation influence recognition by macrophages and other immune cells. Some nanomaterials may themselves stimulate inflammatory responses if their surface chemistry or persistence is unfavorable. Antioxidant capacity must therefore be evaluated together with immunocompatibility.

### 4.4. Cancer: Protection Versus Therapeutic ROS Manipulation

Cancer requires a particularly explicit distinction between antioxidant and pro-oxidant nanotechnology. Nanoantioxidants are intended to decrease damaging oxidative stress, for example by protecting normal tissues from treatment-associated injury. In contrast, nanomaterials deliberately designed to generate ROS within tumors—through Fenton-like chemistry, photodynamic reactions, or other pro-oxidant mechanisms—should be regarded as redox-modulating anticancer nanomaterials rather than as nanoantioxidants in the functional sense [46,47].

This distinction is important because the same material platform may support opposite therapeutic strategies depending on dose, localization, catalytic state, and tumor microenvironment. Antioxidant protection of healthy tissue and intentional ROS amplification in cancer cells should therefore be described as separate objectives, even when both exploit nanoscale control of redox chemistry.

Spatial selectivity is central to reconciling these objectives. Advanced platforms may exploit differences in pH, enzyme expression, redox state, or targeting ligands to confine antioxidant or pro-oxidant activity to selected tissues. Such strategies illustrate programmable redox regulation, but their safety depends on demonstrating that off-target tissues do not experience the opposite redox effect.

### 4.5. Wound Healing and Tissue Regeneration

Controlled ROS concentrations contribute to antimicrobial defense, angiogenesis, cell signaling, and tissue repair, whereas persistent oxidative stress can delay healing. Antioxidant nanoparticles, hydrogels, and nanocomposite dressings have shown promising effects primarily in experimental and preclinical wound models [40,48,49,53]. Their value lies in localized redox regulation combined with moisture control, drug delivery, or structural support, but comparative clinical evidence remains limited.

Antioxidant nanoparticles have consequently been incorporated into hydrogels, wound dressings, scaffolds, and other biomaterial systems. These platforms can provide localized ROS scavenging while maintaining a moist and mechanically supportive environment. Incorporation into hydrogels is particularly attractive because the polymer network can retain nanoparticles at the injury site, control their release, and simultaneously carry antimicrobial or regenerative agents.

Because the desirable redox state changes across stages of wound healing, next-generation systems should favor localized and time-dependent antioxidant activity rather than continuous maximal ROS suppression.

### 4.6. Protection Against Organ and Cellular Oxidative Injury

Beyond specific diseases, nanoantioxidants have been investigated as general cytoprotective agents against oxidative injury in the liver, kidneys, lungs, retina, and other tissues. Their potential is particularly relevant when oxidative stress is spatially localized or occurs rapidly, making conventional systemic antioxidant administration inefficient.

Nanomaterials with regenerative catalytic activity may provide sustained protection without requiring continuous administration of large antioxidant doses. Furthermore, surface functionalization can enhance cellular uptake or promote preferential accumulation in specific tissues. Mitochondria-targeted antioxidant nanomaterials are of particular interest because mitochondria are simultaneously major sources and major targets of cellular ROS [50].

Subcellular targeting represents an important evolution in nanoantioxidant design. Rather than merely increasing total antioxidant concentration, nanoplatforms can potentially position antioxidant activity close to the principal site of ROS generation. This strategy may increase therapeutic efficiency while minimizing disruption of redox signaling elsewhere in the cell.

### 4.7. Antioxidant Nanocarriers and Controlled Delivery

Many natural antioxidants exhibit promising biological activity in vitro but poor therapeutic performance because of low aqueous solubility, chemical instability, rapid metabolism, limited absorption, or nonspecific distribution. Nanocarriers can address these limitations by encapsulating antioxidants within polymeric nanoparticles, liposomes, lipid nanoparticles, micelles, or hybrid structures [51,52].

Nanoencapsulation can protect antioxidant molecules from premature oxidation and degradation, enhance apparent solubility, prolong circulation, and enable controlled release. Surface modification may further provide tissue or cell targeting. Curcumin, resveratrol, quercetin, catechins, vitamins, and other antioxidant compounds have consequently been extensively investigated in nanoscale delivery systems.

Stimuli-responsive nanocarriers provide an additional level of control. Elevated ROS concentrations themselves can serve as triggers for drug release, creating systems in which oxidative stress promotes localized antioxidant delivery. Other triggers include acidic pH, enzymes, temperature, and light. Such responsive systems move antioxidant therapy toward on-demand redox intervention, potentially increasing efficacy while reducing unnecessary exposure.

Across biomedical applications, pharmacokinetics and biological transformation are as important as nominal antioxidant capacity. Circulation time, protein-corona formation, organ distribution, cellular internalization, degradation or dissolution, metabolism of carrier components, and long-term clearance determine both efficacy and toxicity. A formulation that performs well in a cell-free assay may therefore show a different redox profile in vivo after adsorption of biomolecules or transformation in lysosomal, hepatic, or inflammatory environments. Quantitative exposure data should be reported whenever possible to connect administered dose with the material state and concentration present at the biological target.

### 4.8. Theranostics and Precision Redox Medicine

The integration of antioxidant therapy with diagnostic functions is an emerging, largely preclinical direction. Nanomaterials can be engineered to combine ROS regulation with fluorescence, magnetic resonance, photoacoustic, or other imaging capabilities, allowing oxidative environments, nanoparticle localization, or treatment responses to be monitored simultaneously [41,53].

Theranostic platforms could ultimately support treatment adjustment according to local oxidative status, but most current demonstrations remain at proof-of-concept or preclinical stages. Their translation will require evidence that the diagnostic signal accurately reflects the relevant redox process and that the added imaging or responsive components do not compromise pharmacokinetics, safety, or manufacturability.

From a practical perspective, antioxidant-loaded PLGA, lipid, and related carrier formulations have progressed beyond purely chemical proof-of-concept to in vivo pharmacokinetic or disease-model evaluation for conventional antioxidant payloads [5,51,52]. By contrast, most intrinsically redox-active inorganic nanozymes discussed in this review remain supported mainly by in vitro and animal evidence. Across both groups, major barriers include batch-to-batch reproducibility, scalable and quality-controlled manufacturing, sterilization and storage stability, immunotoxicity, chronic retention, regulatory classification, and demonstration of clinically meaningful benefit over existing antioxidants or delivery systems. Biomedical development should therefore prioritize selective, spatially and temporally controlled redox modulation together with quantitative biodistribution, clearance, and long-term safety rather than antioxidant capacity alone.

### 4.9. Evidence Level, Safety, and Clinical Translation

The biomedical literature reviewed above spans very different levels of evidence, and these levels should not be treated as equivalent. Chemical ROS-scavenging or enzyme-like activity provides mechanistic evidence; cell-culture studies demonstrate biological activity under simplified exposure conditions; and animal models provide preclinical information on efficacy, biodistribution, pharmacokinetics, clearance, and tissue-level toxicity. Most nanoantioxidant approaches discussed in this section remain at the chemical, in vitro, or animal proof-of-concept stage. Clinical evidence is much more limited, so terms such as “promising” or “therapeutic” should be interpreted as describing preclinical potential unless human data are specifically available.

Safety cannot be inferred from short-term cell viability or from antioxidant activity alone. Translational assessment should include repeated-dose and long-term toxicity, organ accumulation, biodistribution, biodegradation or persistence, clearance routes, immunological responses, hemocompatibility where relevant, and the possibility that local chemistry converts an antioxidant material into a pro-oxidant. Particle transformations after protein adsorption, lysosomal uptake, dissolution, or metabolic processing should be characterized because the biologically encountered material may differ substantially from the as-synthesized formulation [15,17].

Progress toward clinical translation will also require reproducible manufacturing, validated potency assays, formulation stability, scalable purification, pharmacokinetic and dose optimization, and comparison with existing antioxidant or anti-inflammatory therapies. A favorable chemical assay or a single disease-model result should therefore be regarded as an early step in an evidence chain rather than proof of clinical efficacy. For biomedical nanoantioxidants, the most credible translational claims are those supported by convergent mechanistic, cellular, in vivo, safety, and eventually human evidence.

## 5. Food, Agricultural, and Environmental Applications of Nanoantioxidants

### 5.1. Food Preservation and Oxidative Stability

**Figure 5:**
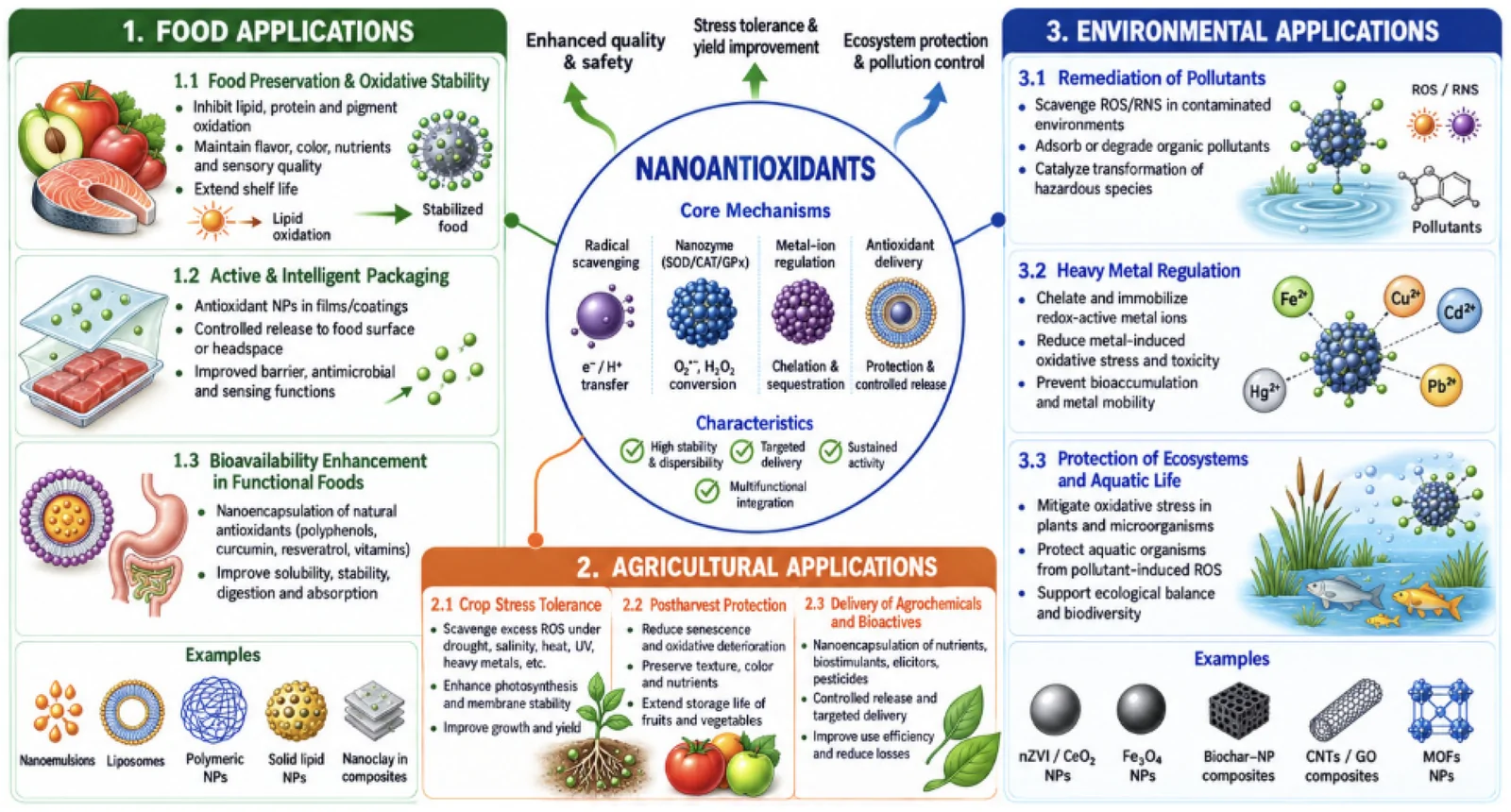
Food, agricultural, and environmental applications of nanoantioxidants [54,56,58,59].

Oxidation is a major cause of food-quality deterioration, affecting lipids, proteins, pigments, vitamins, flavor, color, texture, and shelf life. Nanoencapsulation can protect oxidation-sensitive compounds and improve their dispersion, but performance depends strongly on the food matrix. Lipid content, proteins, polysaccharides, pH, ionic strength, water activity, processing temperature, and interfacial structure can alter nanoparticle stability, antioxidant partitioning, release, and access to oxidation sites [54,55,56,57]. Section 5.1 therefore focuses on matrix-level oxidative stability, while Section 5.2, Section 5.3 and Section 5.6 address packaging, gastrointestinal fate, and postharvest protection, respectively, to minimize overlap among these applications. As summarized in Figure 5, nanoantioxidants provide multifunctional strategies for controlling oxidative processes across food, agricultural, and environmental systems.

Their major functions include improving food oxidative stability and active packaging, enhancing antioxidant bioavailability, protecting crops against abiotic stress, supporting postharvest preservation and controlled agrochemical delivery, and mitigating pollutant- and metal-induced oxidative stress. Collectively, these applications demonstrate how ROS regulation, controlled delivery, and interfacial engineering can contribute to improved product quality, agricultural productivity, environmental protection, and sustainability [54,55,56,57,58,59,60,61,62,63,64,65,66,67].

### 5.2. Active and Intelligent Food Packaging

Active packaging represents one of the most promising technological applications of nanoantioxidants. Unlike conventional packaging, which primarily functions as a passive barrier, active packaging can interact with the packaged environment to inhibit oxidation, microbial growth, moisture accumulation, or other deterioration processes.

Antioxidant nanoparticles or antioxidant-loaded nanocarriers can be incorporated into polymer films, coatings, multilayer structures, and biodegradable packaging materials [58,59]. In active packaging, release toward the food surface or headspace may be desirable, whereas in other designs nanoparticle immobilization is preferred. Actual consumer exposure therefore depends on the food matrix, contact time, temperature, acidity, fat content, mechanical abrasion, and degradation of the packaging, all of which can influence migration of intact particles, dissolved ions, molecular antioxidants, or transformation products.

Multifunctional packaging can combine antioxidant, antimicrobial, barrier, and sensing functions, but migration and consumer safety must be evaluated under realistic food-contact conditions. Assessment should distinguish intentional release of an antioxidant payload from unintended transfer of the nanocarrier or its degradation products. Food-contact applications also require compliance with application-specific regulatory requirements, including migration testing, toxicological evaluation, compositional control, and documentation of exposure. Consequently, improvements in shelf life or packaging performance should be interpreted together with measured migration and safety data rather than as stand-alone indicators of suitability [54,56,57,58,59,60].

### 5.3. Enhancement of Antioxidant Bioavailability in Functional Foods

Many natural antioxidants show strong activity in chemical assays but limited effectiveness after ingestion because their behavior changes during digestion. Food-matrix binding, lipid digestion, pH changes, digestive enzymes, bile salts, and interactions with proteins or minerals can transform both the antioxidant and its nanocarrier. Lipid-based, polymeric, or protein-based nanocarriers may improve solubilization and protect sensitive compounds, but they can also undergo structural reorganization, digestion, or premature release in the gastrointestinal tract [54,60].

It is therefore important to distinguish antioxidant content, bioaccessibility, intestinal absorption, and true systemic bioavailability. Bioaccessibility describes the fraction released from the food matrix and available for intestinal uptake, whereas systemic bioavailability additionally depends on epithelial transport, metabolism, distribution, and elimination. Improved bioaccessibility does not necessarily translate into a proportional increase in circulating or tissue exposure, and higher exposure is not automatically beneficial. Functional-food nanoantioxidants should consequently be evaluated using realistic food matrices, digestion and intestinal models, and, where relevant, pharmacokinetic or in vivo measurements rather than conventional radical-scavenging assays alone.

### 5.4. Crop Protection Against Abiotic Stress

Plants continuously generate ROS as part of normal metabolism and signaling, but environmental stresses can disturb this balance and cause excessive ROS accumulation. Drought, salinity, extreme temperature, high light intensity, heavy metals, and other stressors can increase production of superoxide, hydrogen peroxide, hydroxyl radicals, and related reactive species. These species can damage cellular membranes, photosynthetic machinery, proteins, pigments, and nucleic acids.

Plant exposure is also governed by the application route and the surrounding biological environment. Root uptake and foliar uptake can lead to different translocation pathways, while interactions with the rhizosphere, root exudates, and soil microbiota may transform nanoparticles before they reach plant tissues. Repeated application raises additional questions regarding accumulation in soil, persistence, transfer to edible tissues, trophic exposure, and effects on beneficial microorganisms. Agronomic efficacy should therefore be evaluated together with uptake, translocation, transformation, and residue measurements across realistic soil–plant systems, particularly when large-scale or repeated use is envisioned [61,62].

### 5.5. Nanotechnology-Assisted Delivery of Plant Antioxidants and Agrochemicals

Nanocarriers can also be used to deliver bioactive compounds that support plant health. Conventional agricultural chemicals often suffer from poor water solubility, rapid degradation, nonspecific distribution, and substantial losses to soil or water. Nanoscale delivery systems can potentially improve retention, controlled release, and targeting while reducing the total quantity of active material required [62,63].

Polymeric nanoparticles, lipid-based carriers, mesoporous materials, and biogenic nanostructures can improve retention and controlled release of antioxidants, nutrients, elicitors, or agrochemicals [62,63]. For practical agricultural use, however, improved delivery efficiency must be balanced against persistence of the carrier, transformation in soil and water, interactions with non-target organisms, and the possibility of repeated accumulation. Demonstrating lower total chemical input is therefore not sufficient unless the environmental fate of the nanoscale delivery system is also characterized.

### 5.6. Postharvest Protection and Shelf-Life Extension

Postharvest applications differ from functional-food delivery because their primary goal is to preserve harvested tissues during storage rather than to enhance systemic bioavailability after ingestion. Nanoenabled edible coatings and packaging can limit oxygen and moisture transfer and can provide localized release of antioxidant or antimicrobial agents [56,58]. Their performance should therefore be evaluated using shelf-life, sensory, migration, and surface-exposure endpoints rather than repeating the delivery criteria used for ingested nanocarriers.

Controlled antioxidant release may delay pigment loss, membrane deterioration, lipid oxidation, and microbial spoilage. Nevertheless, the formulation must remain compatible with sensory quality and food-contact requirements, and any migration of nanoparticles or degradation products should be considered when assessing consumer exposure and end-of-life impacts.

### 5.7. Environmental Remediation and Protection

Environmental use encompasses two related but distinct objectives. In organism-protection applications, a nanoantioxidant is applied to reduce oxidative stress in plants, microorganisms, or aquatic organisms exposed to environmental stressors. In remediation applications, by contrast, a nanomaterial is used directly to immobilize metals, adsorb pollutants, or catalyze contaminant transformation [64,65]. The latter function is not necessarily antioxidant in a biological sense and should be evaluated according to contaminant removal, reaction products, and ecological safety as well as changes in ROS.

For remediation, surface-functionalized nanoparticles may bind redox-active metal ions or adsorb organic contaminants, thereby reducing mobility or modifying pollutant-driven oxidative pathways. However, catalytic transformation of contaminants can also generate reactive intermediates. The environmental benefit therefore depends on the complete reaction pathway and on the fate of both the pollutant and the nanomaterial.

Environmental transformations are especially important because nanoparticles released into soils or natural waters can aggregate, dissolve, oxidize or reduce, adsorb natural organic matter, acquire environmental coronas, or undergo surface modification. These processes can change mobility, bioavailability, antioxidant or pro-oxidant activity, and ecological toxicity. Environmental nanoantioxidants should therefore be tested across relevant transformation states rather than only as freshly synthesized particles, and organism-protection claims should be clearly separated from direct remediation functions.

### 5.8. Sustainability, Safety, and Life-Cycle Considerations

The potential sustainability advantages of nanoantioxidants include reduced antioxidant consumption, extended food shelf life, lower food waste, improved agricultural stress tolerance, and more efficient use of bioactive compounds. However, “green synthesis” should describe a synthesis route rather than serve as a surrogate for overall sustainability. Use of plant extracts, polysaccharides, proteins, microorganisms, or other renewable reagents may reduce reliance on some hazardous chemicals [66,67], but the environmental benefit depends on the complete process.

A credible sustainability assessment should consider precursor sourcing and cultivation, solvent and water consumption, reaction temperature and time, energy demand, purification and washing steps, yield, batch reproducibility, scale-up, waste generation, nanoparticle release during use, recyclability, and end-of-life fate. For biogenic synthesis in particular, repeated centrifugation, dialysis, drying, sterilization, or low-yield purification can offset advantages associated with a renewable reducing agent. Life-cycle assessment and mass/energy balances are therefore preferable to categorical claims that a nanomaterial is sustainable simply because a biological or lower-hazard synthesis route was used.

Safety and sustainability should therefore be evaluated across the complete life cycle. Relevant endpoints include migration and ingestion exposure, gastrointestinal transformation, soil and water fate, biodegradation or persistence, dissolution and ion release, bioaccumulation, ecological effects, and options for recovery, recycling, or safe disposal. Green synthesis may improve selected production metrics but does not substitute for this broader assessment. Future food, agricultural, and environmental nanoantioxidants should combine effective redox control with predictable transformation, low toxicity, scalable production, and demonstrable life-cycle advantages over conventional alternatives.

## 6. Materials, Industrial, and Engineering Applications of Nanoantioxidants

**Figure 6:**
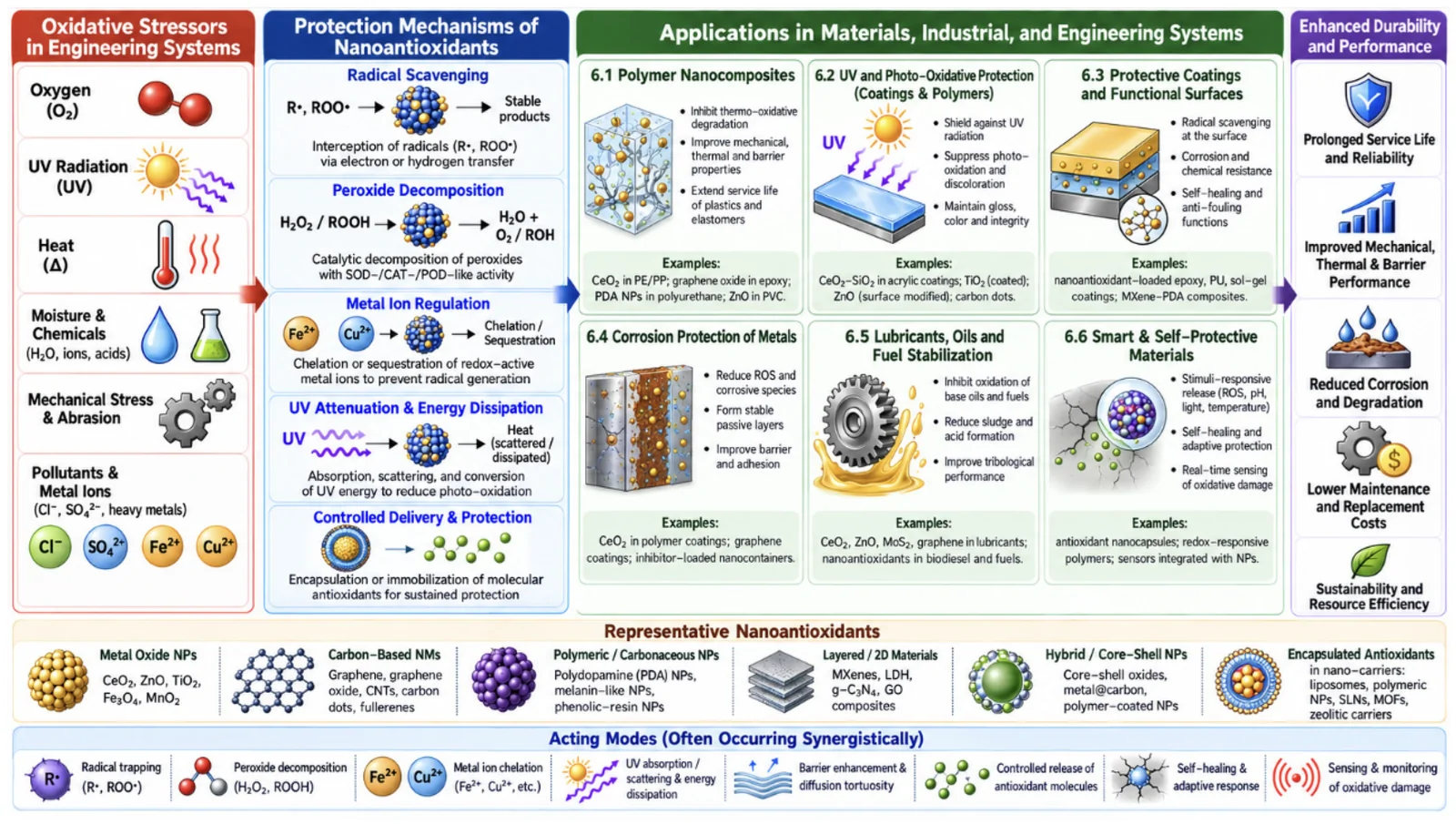
Materials, Industrial, and Engineering Applications of Nanoantioxidants [68,69,70].

Oxidative degradation is not restricted to biological systems but also represents a major cause of deterioration in polymers, coatings, lubricants, fuels, construction materials, and other engineered products. Exposure to oxygen, ultraviolet (UV) radiation, elevated temperature, moisture, pollutants, and mechanical stress can initiate radical reactions that progressively alter molecular structure and compromise material performance. Conventional antioxidants and stabilizers are widely employed to interrupt these processes; however, their effectiveness can be limited by volatility, migration, leaching, thermal degradation, incompatibility with the host matrix, and depletion during long-term exposure. Nanoantioxidants provide an alternative strategy by combining high interfacial area, tunable surface chemistry, catalytic redox activity, UV attenuation, and the controlled immobilization or delivery of antioxidant compounds. These characteristics enable nanoantioxidants to function not only as chemical stabilizers but also as multifunctional components capable of simultaneously improving barrier, mechanical, thermal, optical, and protective properties [68,69,70]. As illustrated in Figure 6, nanoantioxidants can mitigate oxidative degradation in engineering systems through complementary mechanisms, including radical scavenging, peroxide decomposition, metal-ion regulation, UV attenuation, and controlled antioxidant delivery. These mechanisms can be integrated into polymer nanocomposites, protective coatings, corrosion-control systems, lubricants, and smart self-protective materials to improve durability, reduce degradation, and extend service life [68,69,70,71,72,73,74,75,76,77,78,79,80,81].

Industrial implementation introduces constraints that are not captured by laboratory antioxidant assays. Large-scale synthesis must provide consistent particle size, surface chemistry, redox state, and purity from batch to batch; nanoparticles must remain dispersible during mixing, extrusion, coating, curing, or fluid formulation; and the additive must be compatible with existing manufacturing equipment and quality-control procedures. Cost, storage stability, worker exposure, process yield, and the ability to maintain performance after thermal and mechanical processing are therefore central criteria for evaluating whether a nanoantioxidant can progress beyond laboratory-scale demonstrations.

### 6.1. Oxidative Stabilization of Polymeric Materials

Polymeric materials are particularly susceptible to oxidative aging. Thermal energy, UV irradiation, mechanical stress, and environmental contaminants can generate macroradicals that react rapidly with oxygen to form peroxyl radicals and hydroperoxides. Their subsequent decomposition generates additional reactive intermediates, resulting in a self-propagating oxidation cycle. Chain scission, crosslinking, formation of oxygen-containing functional groups, discoloration, embrittlement, and loss of mechanical properties may consequently occur.

Nanoantioxidants can interrupt this degradation cycle through radical scavenging, peroxide decomposition, metal-ion regulation, or protection of conventional antioxidant molecules. Carbon-based nanomaterials, metal and metal-oxide nanoparticles, and antioxidant-functionalized polymeric nanoparticles have therefore been investigated as stabilizing additives in polymer matrices [68,71].

One important advantage of nanoscale stabilizers is their relatively large interfacial area. When adequately dispersed, a comparatively small amount of nanomaterial can interact with a large fraction of the surrounding polymer matrix. Surface functionalization can further introduce phenolic, catechol, amine, or other redox-active groups capable of intercepting radical intermediates.

However, dispersion is critical. Agglomerated nanoparticles can create local stress concentrations, reduce the accessibility of active surfaces, alter optical properties, and potentially promote rather than inhibit degradation. Effective polymer stabilization therefore requires optimization of nanoparticle concentration, surface compatibility, dispersion, and interfacial bonding.

### 6.2. UV and Photo-Oxidative Protection

Solar UV radiation is a major driver of aging in polymers, coatings, textiles, packaging, and outdoor infrastructure. Absorption of sufficiently energetic photons can produce excited molecular states and radicals, initiating photo-oxidative chain reactions. The resulting chemical changes cause discoloration, surface cracking, loss of gloss, embrittlement, and deterioration of mechanical performance.

Nanomaterials can mitigate photo-oxidation through several complementary mechanisms. Certain inorganic nanoparticles absorb or scatter UV radiation, thereby decreasing the amount of high-energy radiation reaching susceptible polymer chains. Other nanomaterials scavenge ROS generated during irradiation or suppress radical propagation. Combining UV attenuation with antioxidant activity can therefore provide more comprehensive photoprotection than either mechanism alone [69,72].

CeO_2_-based materials are particularly interesting because they combine strong UV absorption with redox-active surfaces. Their performance nevertheless depends on particle size, surface oxidation state, dispersion, and coating chemistry. Similar considerations apply to other semiconductor or metal-oxide nanoparticles. Some UV-absorbing materials may become photocatalytically active and generate ROS under illumination, which would accelerate polymer degradation rather than suppress it.

Surface modification is therefore essential when potentially photocatalytic nanoparticles are incorporated into oxidation-sensitive materials. Coatings, dopants, polymer grafts, or core–shell architectures can suppress undesirable photocatalytic reactions while retaining UV-screening properties.

### 6.3. Protective Coatings and Functional Surfaces

Protective coatings constitute an important engineering platform for nanoantioxidants because oxidative degradation frequently begins at material surfaces exposed directly to oxygen, radiation, moisture, and aggressive chemicals. Incorporating nanoantioxidants into coatings can establish a localized protective layer capable of intercepting ROS and radicals before extensive substrate degradation occurs.

Antioxidant nanomaterials can be incorporated into polymeric, sol–gel, hybrid organic–inorganic, or waterborne coatings. Their functions may include radical scavenging, UV attenuation, inhibition of oxidative chain reactions, corrosion protection, and stabilization of the coating matrix. Nanoparticle incorporation may additionally modify coating hardness, adhesion, permeability, hydrophobicity, and resistance to abrasion.

A particularly promising approach involves immobilizing antioxidant functionality at or near the coating surface. This strategy can decrease migration and volatilization compared with low-molecular-weight antioxidants while maintaining activity where oxidative exposure is greatest. Antioxidant-functionalized nanoparticles may therefore provide longer-lasting protection during environmental aging [73,74].

Multifunctional coatings can further integrate antioxidant activity with antimicrobial, self-healing, anti-corrosion, or sensing functions. For example, stimuli-responsive nanocontainers may release antioxidant or corrosion-inhibiting agents when local pH, moisture, ROS concentration, or mechanical damage changes. Such systems shift protective coatings from passive barriers toward responsive surface-protection technologies.

### 6.4. Corrosion and Oxidative Degradation of Metals

Metal corrosion is fundamentally associated with electrochemical oxidation–reduction reactions and is frequently accelerated by oxygen, moisture, aggressive ions, and local chemical heterogeneity. Although the mechanisms differ from oxidative degradation of organic polymers, ROS and redox-active species can contribute to corrosion processes in certain environments.

Nanomaterials can improve corrosion resistance by strengthening barrier properties, reducing transport of oxygen and aggressive ions, passivating active sites, or delivering corrosion inhibitors. Antioxidant functionality provides an additional strategy in environments where reactive oxygen species contribute to interfacial degradation [73,75].

The challenge is to obtain this electrochemical benefit without compromising coating integrity. Excessive loading or poor dispersion can increase porosity and create transport pathways, while inappropriate redox activity may alter interfacial electrochemistry. Corrosion-protective nanoantioxidants should therefore be assessed as part of the complete coating–substrate system using barrier, adhesion, aging, and electrochemical measurements under relevant environments.

### 6.5. Lubricants, Oils, and Fuel Stabilization

Oxidative deterioration is also a major concern in lubricants, edible and industrial oils, fuels, and other hydrocarbon-based fluids. Elevated temperatures and contact with oxygen can initiate radical oxidation, producing hydroperoxides, acids, sludge, deposits, and other degradation products. These changes increase viscosity, reduce lubrication efficiency, and shorten service life.

Nanoadditives can improve oxidation resistance in lubricants and oils by scavenging radicals, decomposing peroxides, or interacting with catalytic metal species [76,77]. These antioxidant effects should be evaluated separately from tribological functions. A nanoparticle that reduces friction or wear through rolling, polishing, tribofilm formation, or surface repair may improve lubricant performance even if its direct antioxidant contribution is modest; conversely, strong radical-scavenging activity does not guarantee acceptable friction, filtration, or sedimentation behavior.

For lubricants, meaningful evaluation should therefore combine oxidation-induction or aging measurements with friction, wear, viscosity, dispersion stability, filtration compatibility, and interactions with conventional additive packages. This separation of mechanisms is important when attributing a service-life improvement specifically to antioxidant action.

Fuel applications require additional caution because nanoparticles can influence not only storage oxidation but also atomization, combustion kinetics, deposits, emissions, catalyst performance, and fuel-system compatibility. An apparent antioxidant benefit during storage should therefore be evaluated together with combustion efficiency, particulate or gaseous emissions, material compatibility, sedimentation, and downstream effects on engines or after-treatment systems under realistic operating conditions.

### 6.6. Nanocomposites with Combined Antioxidant and Structural Functions

A major technological advantage of nanoantioxidants is the possibility of combining redox regulation with structural reinforcement. Nanoparticles incorporated into polymers can simultaneously influence mechanical properties, barrier performance, thermal stability, electrical conductivity, optical behavior, and resistance to oxidative aging.

Carbon-based nanomaterials provide a representative example. Graphene derivatives, carbon nanotubes, carbon dots, and related structures may improve mechanical or barrier properties while their electronic structure and surface functionality contribute to radical-scavenging or redox behavior. Similarly, inorganic nanoparticles can combine antioxidant or UV-protective activity with stiffness, thermal stability, and barrier enhancement [70,71].

Hybrid nanocomposites can further combine multiple antioxidant mechanisms. A polymer matrix may contain a UV-screening inorganic phase, a radical-scavenging carbonaceous component, and encapsulated molecular antioxidants. Such hierarchical systems can provide complementary protection against initiation, propagation, and secondary oxidative reactions. Nevertheless, increasing compositional complexity also complicates manufacturing, recycling, toxicity assessment, and interpretation of degradation mechanisms. Multifunctional design should therefore prioritize functions that provide measurable improvements in service performance rather than maximizing the number of incorporated components.

### 6.7. Smart and Self-Protective Materials

Smart and self-protective materials should be divided conceptually into demonstrated active-protection strategies and more speculative adaptive concepts. Demonstrated approaches include inhibitor-containing or CeO_2_-based nanocontainers that respond to local corrosion conditions and provide active protection [75], while established autonomic and self-healing polymer concepts provide engineering architectures that can be combined with antioxidant functionality [78,79]. By contrast, fully autonomous systems that sense ROS quantitatively, diagnose remaining antioxidant capacity, and adjust release in closed loop remain an emerging research direction.

Stimuli-responsive carriers can store antioxidants or corrosion inhibitors and release them in response to pH, moisture, redox changes, or mechanical damage. Evidence for such systems should include trigger specificity, release kinetics, retained activity after storage, and repeated-response capability rather than relying only on the presence of a responsive chemistry.

Self-healing architectures offer a complementary route because mechanical damage creates pathways for oxygen, moisture, and aggressive species. Combining a demonstrated healing mechanism with antioxidant or inhibitor release could restore physical barrier integrity while suppressing chemical degradation, but the contribution of each function should be separated experimentally.

Self-reporting concepts use optical, electrical, or fluorescence changes to indicate oxidative aging or depletion of protective agents. Such sensing functions are promising, but direct evidence that the signal quantitatively tracks material degradation under realistic service conditions is still limited. Future work should therefore distinguish proof-of-concept sensing from validated condition-monitoring performance and should test these systems over repeated ageing and damage cycles.

### 6.8. Durability, Safety, and Life-Cycle Design

For engineering applications, the key endpoint should be durable improvement in service performance rather than short-term radical-scavenging capacity. Accelerated tests involving UV radiation, temperature, oxygen, humidity, chemicals, and mechanical loading are useful only when their relationship to realistic field aging is established. Long-term validation should track both material properties and nanoantioxidant transformation so that performance loss can be linked to oxidation-state changes, aggregation, migration, depletion, or interfacial damage.

The durability of the nanoantioxidant itself is equally important. Nanoparticles may undergo oxidation-state changes, dissolution, aggregation, surface contamination, or migration during service. These transformations can alter both antioxidant effectiveness and environmental behavior. Characterization before and after aging is therefore essential for identifying the mechanisms responsible for long-term performance.

Potential nanoparticle release during manufacturing, service, weathering, abrasion, repair, recycling, and disposal must be incorporated into engineering assessment [80,81]. Immobilization in a matrix can reduce initial exposure, but aging or mechanical damage may release intact particles, dissolved species, or transformed products. Recycling routes should therefore be evaluated for their ability to retain, separate, recover, or safely manage nanoadditives rather than assuming that improved durability automatically produces an environmental benefit.

Life-cycle assessment is particularly important because longer service life can reduce replacement frequency, raw-material consumption, maintenance, waste, and associated energy demand, while nanoparticle synthesis, purification, incorporation, and end-of-life treatment can add new burdens. A genuine technological advantage should therefore be demonstrated through a balance of service-life extension, manufacturing impacts, release potential, recyclability, and disposal requirements rather than through antioxidant performance alone.

The engineering value of nanoantioxidants will ultimately depend on reproducible manufacturing, compatibility with industrial processing, realistic long-term aging performance, controllable release, safe end-of-life pathways, and favorable life-cycle trade-offs. Standardized service-relevant testing that combines chemical, mechanical, electrochemical, and environmental endpoints is therefore more informative than isolated short-term antioxidant assays for determining whether these systems provide practical advantages over conventional stabilizers.

## 7. Challenges and Future Perspectives

Despite substantial progress, comparison among nanoantioxidant studies remains difficult because synthesis route, purification, surface functionalization, particle-size distribution, oxidation state, aggregation, dose expression, test medium, exposure duration, and biological model are often reported inconsistently. Harmonized protocols should therefore define a minimum characterization and reporting set that includes primary and hydrodynamic size, surface charge, composition and redox state, surface chemistry, aggregation behavior in the test medium, concentration expressed on appropriate mass/molar/surface-area bases, exposure conditions, and the exact analytical endpoint. Without this information, apparent differences in antioxidant efficacy may reflect preparation or measurement artifacts rather than genuine structure–activity relationships. More fundamentally, the central challenge is not simply to determine whether a nanomaterial can scavenge ROS, but to establish whether its redox response can be predicted and controlled across realistic operating environments. Future studies should therefore define multidimensional activity windows that simultaneously consider dose, exposure time, pH, ionic strength, temperature, biomolecular corona formation, and competing oxidants or reductants. Such studies would help convert empirical observations into mechanistic design rules linking physicochemical descriptors to antioxidant or pro-oxidant outcomes.

Standardization should also distinguish chemical scavenging assays, catalytic kinetic measurements, cell-based oxidative-stress endpoints, and in vivo outcomes. Orthogonal methods, appropriate nanoparticle-only controls, reference materials, and transparent reporting of negative or inconclusive results would improve reproducibility and interlaboratory comparison [20]. For biomedical and food applications, dose and exposure duration must be linked to biodistribution, gastrointestinal or biological transformation, clearance, and possible long-term accumulation. Short-term antioxidant efficacy should not be assumed to imply safety during repeated or chronic exposure. In the authors’ view, standardization should extend beyond assay selection to include reference materials, orthogonal validation methods, kinetic measurements, and explicit reporting of nanoparticle concentration on mass-, molar-, and surface-area-normalized bases where appropriate. Direct comparison with established molecular antioxidants would also provide a more meaningful benchmark of whether nanoscale formulations offer a genuine functional advantage. Without such harmonization, differences among studies may reflect methodological artifacts rather than intrinsic material performance.

Safety and long-term fate therefore require application-specific assessment. Biomedical development should address immunological interactions, organ distribution, degradation, clearance, and chronic toxicity; food applications should consider repeated ingestion, digestion products, and tissue exposure; and agricultural, environmental, and engineering uses should evaluate migration, persistence, transformation, and release during use and disposal. Safe-by-design strategies should distinguish biodegradable, transformable, and persistent nanoantioxidants and should incorporate these differences before performance optimization is finalized.

A second translational gap is the transition from simplified laboratory systems to complex real-world environments. Activity measured in buffer or cell culture may change in blood, gastrointestinal fluids, heterogeneous foods, soils, natural waters, coatings, lubricants, or weathered polymer matrices because the nanoparticle surface and surrounding reactants are different. Future studies should therefore progress from mechanistic screening to in vivo validation, realistic exposure simulations, long-duration field or service tests, and pilot-scale manufacturing. Demonstrating robustness across these levels is necessary before laboratory antioxidant performance can be treated as evidence of practical effectiveness.

Future research should focus on selective and responsive redox regulation rather than simply maximizing ROS-scavenging activity. Targeted and stimuli-responsive nanomaterials could provide antioxidant activity preferentially at sites of excessive oxidative stress while preserving physiological ROS signaling. Biodegradable carriers, multifunctional nanozymes, and environmentally responsive systems are particularly promising for achieving this objective. This shift can be viewed as a move from passive ROS scavenging toward precision redox engineering. Because physiological and technological systems often require a finite level of ROS for signaling, antimicrobial defense, curing reactions, or other functions, complete and persistent ROS suppression may be undesirable. An important long-term objective is therefore to engineer threshold-triggered or feedback-responsive systems that become active only when oxidative stress exceeds a defined level and subsequently deactivate or degrade when redox balance is restored.

Machine learning and artificial intelligence may assist structure–activity analysis and candidate prioritization, but their role should remain subordinate to experimental validation. Useful models should integrate not only antioxidant activity but also toxicological, biodistribution, persistence, environmental, processing, and life-cycle endpoints so that optimization does not favor redox performance at the expense of safety or sustainability. Model reliability will depend on standardized, interoperable datasets that include negative results, synthesis history, uncertainty, and test-condition metadata. Prospective experimental validation and mechanistic interpretation are essential before computationally identified candidates can be considered credible design solutions.

The author proposes that future progress can be organized into three complementary horizons. In the near term, priority should be given to reproducible synthesis, standardized characterization, assay harmonization, and transparent reporting. In the medium term, research should emphasize safe-by-design optimization, realistic long-term validation, pilot-scale manufacturing, and sector-specific regulatory requirements. In the longer term, the field should move toward adaptive and biodegradable redox systems supported by digital materials design, real-time sensing, and closed-loop optimization. Importantly, success should be judged against conventional antioxidants and existing technologies using integrated criteria that include efficacy, selectivity, toxicity, durability, cost, energy demand, and end-of-life impact rather than ROS-scavenging capacity alone.

**Figure 7:**
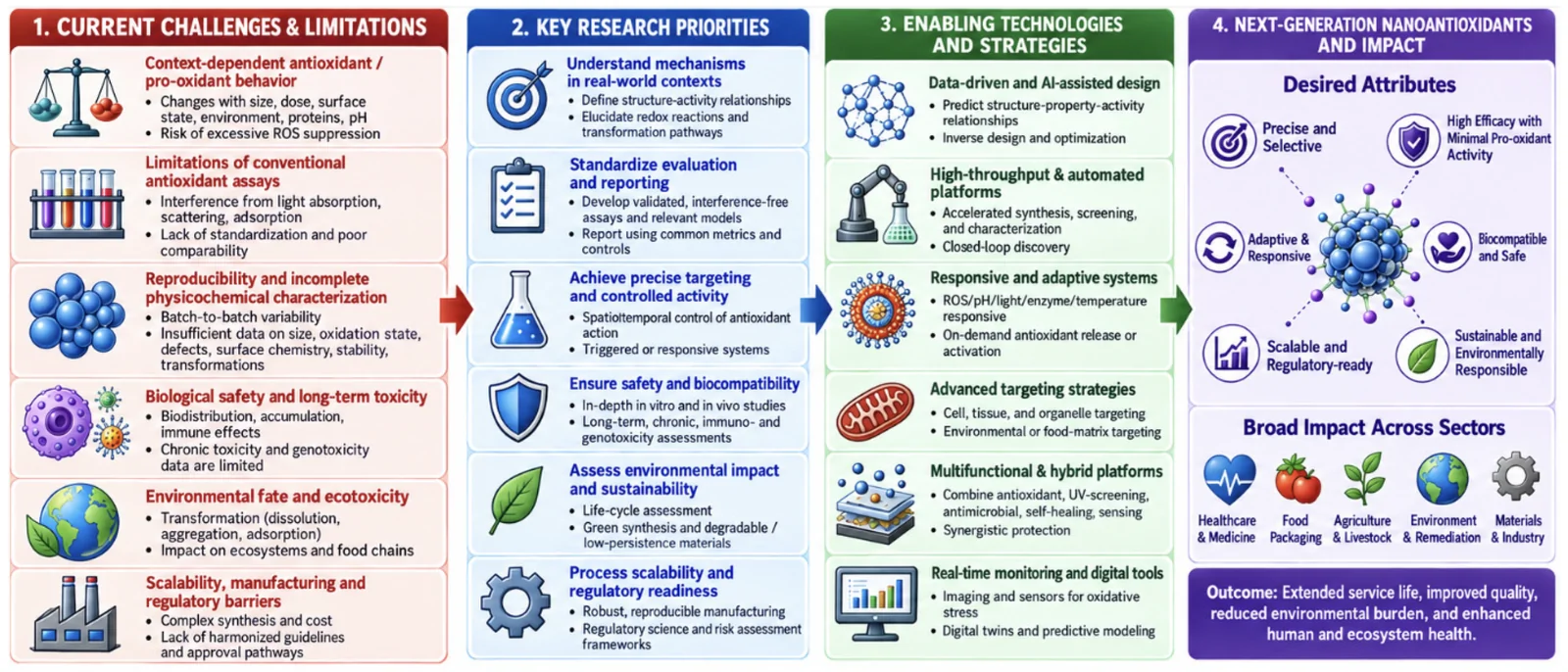
Roadmap for the Development of Next-Generation Nanoantioxidants.

Successful translation will ultimately be determined by whether nanoantioxidants can be manufactured reproducibly and economically, incorporated into existing application workflows, validated under realistic long-term conditions, accepted within relevant regulatory frameworks, and shown to provide a favorable life-cycle benefit. Figure 7 summarizes this translational roadmap. The decisive shift for the field is therefore from demonstrating high antioxidant activity under idealized laboratory conditions to establishing predictable, application-specific, scalable, and life-cycle-validated redox performance.

Regulatory considerations represent a major challenge in translating nanoantioxidants from laboratory research to commercial products. Unlike conventional antioxidants, nanomaterials may exhibit size-, surface-, and structure-dependent biological behaviors that cannot always be adequately evaluated using conventional safety assessment frameworks. Regulatory evaluation therefore requires careful characterization of physicochemical properties, including particle size distribution, surface chemistry, aggregation state, composition, stability, and potential transformation during storage, use, and disposal. Particular attention should also be given to dose-dependent toxicity, biodistribution, persistence, degradation products, and possible long-term effects. The regulatory pathway is strongly dependent on the intended application. Nanoantioxidants developed for biomedical, food, agricultural, cosmetic, or environmental applications may be subject to different requirements concerning toxicity testing, exposure assessment, manufacturing quality, labeling, and environmental risk. This diversity makes harmonization of testing protocols and safety criteria particularly important for facilitating commercialization across different sectors and jurisdictions. Establishing standardized characterization methods, validated toxicity models, reproducible manufacturing procedures, and transparent reporting of nanomaterial properties would therefore reduce regulatory uncertainty and improve comparability among studies. Future commercialization will require regulatory considerations to be incorporated at an early stage of material design rather than addressed only after performance optimization. A safe-by-design approach, integrating efficacy, safety, manufacturability, environmental fate, and regulatory compliance, could accelerate the transition of nanoantioxidants from proof-of-concept studies to practical products. Close collaboration among researchers, manufacturers, toxicologists, regulatory authorities, and end users will consequently be essential for developing scientifically robust and commercially viable nanoantioxidant technologies.

## 8. Conclusions

Nanoantioxidants should be understood as a family of intrinsically redox-active nanomaterials, antioxidant nanocarriers, and hybrid systems whose performance is governed by structure, surface state, exposure conditions, and biological or technological context. The central conclusion of this review is that antioxidant activity is not an intrinsic or universally beneficial property of a nanomaterial. Changes in dose, oxidation state, surface chemistry, aggregation, localization, dissolution, and surrounding environment can shift the same platform from antioxidant toward ineffective or pro-oxidant behavior.

The breadth of reported biomedical, food, agricultural, environmental, and engineering applications demonstrates substantial potential, but it also highlights the gap between promising laboratory results and practical translation. Most intrinsically redox-active nanozymes remain at the laboratory or preclinical stages, and broader implementation will require reproducible manufacturing, scalable processing, realistic long-term testing, quantitative biodistribution or environmental-fate information, and application-specific regulatory evaluation. Performance should therefore be judged against existing antioxidants and technologies rather than by radical-scavenging capacity alone.

Nevertheless, antioxidant performance is strongly dependent on particle composition, size, morphology, oxidation state, defect structure, surface chemistry, aggregation, dissolution, dose, and surrounding conditions. The same nanomaterial may shift from antioxidant to pro-oxidant behavior when pH, concentration, metal-ion release, light exposure, biomolecular adsorption, or other environmental variables change. Reliable interpretation therefore requires nanoparticle-specific controls, complementary assays, explicit separation of chemical, cellular, animal, and clinical evidence, and comprehensive evaluation of long-term safety, biodistribution or environmental fate, degradation, and life-cycle impacts.

Future research should prioritize precise and adaptive redox regulation rather than maximum antioxidant capacity in a single assay. Mechanism-guided structure–activity studies, reproducible manufacturing, standardized metadata, safe-by-design principles, realistic long-term validation, and AI-assisted optimization based on harmonized experimental datasets may accelerate progress. For biogenic systems, the contribution of residual phytochemicals should be separated from intrinsic nanoparticle activity; for “green” synthesis, sustainability should be supported by process and life-cycle evidence; and for biomedical applications, preclinical promise should be distinguished clearly from clinical efficacy. Balancing efficacy, selectivity, safety, scalability, and sustainability will be essential for translating nanoantioxidants into reliable technologies for human health, food security, environmental protection, and durable materials.

## Figures and Tables

**Table 1:** Hierarchical classification, functional role, representative materials, design characteristics, antioxidant mechanisms, advantages, and major limitations of nanoantioxidant platforms.

Nanoantioxidant Class	Representative Materials	Key Design Parameters	Principal Antioxidant Mechanisms	Major Advantages	Key Limitations	Refs.
Intrinsic nanoantioxidants—metal/metal-oxide (umbrella class)	CeO_2_, Mn_3_O_4_, Pt nanoparticles	Oxidation state; oxygen vacancies; crystal facets; particle size; surface coating	Superoxide Dismutase (SOD)-/Catalase (CAT)-like activity; catalytic ROS decomposition; regenerative redox cycling	High catalytic activity; potentially regenerative; tunable surface redox chemistry	Possible pro-oxidant activity; aggregation; persistence; strong environmental dependence	[7,8,9,10,11,12,13,14,15,16]
Metal/metal-oxide subtype—CeO_2_-based nanoantioxidants	Nanoceria; polyethylene glycol (PEG)ylated CeO_2_	Ce^3+^/Ce^4+^ ratio; vacancy density; surface Ce^3+^ concentration; PEGylation; surrounding ions	SOD- and CAT-mimetic activity; radical scavenging; Ce^3+^↔Ce^4+^ cycling	Multienzyme-like activity; regenerative antioxidant function; tunable chemistry	Phosphate/environment-dependent activity; surface-state changes; concentration-dependent effects	[4,7,8,9,10,11,13,16,17,18,19,20]
Metal/metal-oxide subtype—Mn-based nanozymes	Mn_3_O_4_ nanoparticles	Mn oxidation states; size; morphology; accessible catalytic sites	SOD-, CAT-, and Glutathione Peroxidase (GPx)-like activities; broad ROS regulation	Multiple enzyme-mimetic functions within one nanostructure	Activity depends on surface state, dose, and biological environment	[12]
Metal/metal-oxide subtype—noble-metal nanoantioxidants	Pt nanoparticles	Particle size; surface area; ligand chemistry; dispersion	Scavenging/decomposition of O_2_•^−^ and H_2_O_2_; enzyme-mimetic catalysis	Strong catalytic activity; high chemical stability	Cost; persistence; surface-dependent biological interactions	[21,22]
Intrinsic nanoantioxidants—carbon-based	Fullerenes; functionalized fullerenes; graphene derivatives; carbon dots	Conjugated structure; defects; heteroatom doping; surface functionalization; dispersibility	Radical addition; electron transfer; radical stabilization; ROS scavenging	Tunable electronic structure; multifunctionality; potential imaging capability	Aggregation; variable biocompatibility; possible context-dependent pro-oxidant effects	[6,23,24]
Antioxidant nanocarriers—polymeric	Poly(lactic-co-glycolic acid) (PLGA) nanoparticles; chitosan-based nanoparticles	Polymer composition; particle size; loading efficiency; degradation rate; surface functionalization	Protection and controlled release of molecular antioxidants	Improved solubility, stability, bioavailability, and controlled delivery	Limited intrinsic catalytic activity; formulation-dependent release; degradation considerations	[5,25]
Antioxidant nanocarriers—lipid-based	Liposomes; solid lipid nanoparticles; nanostructured lipid carriers	Lipid composition; particle size; membrane properties; antioxidant loading	Encapsulation and protection of antioxidants; controlled delivery	Suitable for lipophilic antioxidants; good loading capability	Oxidative instability of lipid components; leakage; storage stability	[26]
Intrinsic nanoantioxidants—redox-active polymeric nanoparticles	Polydopamine nanoparticles	Catechol/quinone ratio; particle size; specific surface area; degree of oxidation	Electron/H-atom transfer; direct ROS scavenging; redox cycling	Intrinsic antioxidant activity; surface functionalization; multifunctional platform	Activity strongly depends on oxidation state and nanostructure	[27,28]
Biogenic nanoantioxidants	Plant-mediated metal/metal-oxide nanoparticles	Biological precursor; phytochemical composition; synthesis conditions; surface capping	Intrinsic nanoparticle redox activity plus phytochemical radical scavenging	Potentially greener synthesis; biologically derived surface functionality	Batch variability; difficult standardization; complex surface composition	[29,30]
Hybrid/multifunctional systems	Metal–polymer; metal oxide–carbon; biomolecule–inorganic hybrids	Component ratio; interfacial structure; architecture; functional integration	Combined catalytic ROS decomposition, radical scavenging, delivery, and responsive activity	Multiple complementary mechanisms; potential targeting and theranostic functions	Greater synthesis/characterization complexity; reproducibility and scale-up challenges	[31]

**Table 2:** Major Antioxidant Mechanisms of Nanomaterials and Their Functional Characteristics.

Mechanism	Representative Platforms	Key Reaction/ Process	Main ROS/Targets	Functional Significance	Key Determinants/Limitations	Refs.
Direct radical scavenging	Carbon-based nanomaterials; polydopamine; functionalized nanoparticles	Electron transfer (ET), H-atom transfer (HAT), proton-coupled ET, or radical addition	O_2_•^−^, •OH, ROO• and other radical intermediates	Interrupts radical-chain reactions and limits oxidative damage to lipids, proteins and nucleic acids	Depends on accessible surface groups, specific surface area, oxidation state, aggregation and passivation	[32,35,36]
SOD-like activity	Ce-, Mn- and other transition-metal nanozymes	2O_2_•^−^ + 2H^+^ → H_2_O_2_ + O_2_	Superoxide (O_2_•^−^)	Catalytically converts superoxide to H_2_O_2_ and O_2_; enables repeated ROS regulation	Controlled by surface oxidation states, defects, coordination environment, particle size and facets	[32,33,34]
CAT-like activity	Metal/metal-oxide nanozymes; multienzyme-mimetic nanostructures	2H_2_O_2_ → 2H_2_O + O_2_	Hydrogen peroxide (H_2_O_2_)	Removes H_2_O_2_ and complements SOD-like activity in sequential ROS detoxification	Strongly affected by oxidation state, pH, substrate concentration, surface accessibility and local environment	[32,33,34]
POD-like activity	Metal-containing nanozymes	Catalytic peroxide conversion using reducing substrates	H_2_O_2_ and electron-donor substrates	Can assist peroxide removal, but the net effect depends on the complete catalytic pathway	May become pro-oxidant through Fenton/Fenton-like •OH generation; activity is pH- and substrate-dependent	[32,34]
GPx-like activity	Se-, Te-, metal- and carbon-containing nanozymes; multi-nanozyme systems	Reduction of H_2_O_2_ or organic hydroperoxides using reducing equivalents/thiols	H_2_O_2_ and organic hydroperoxides	Limits peroxide accumulation and lipid oxidation; complements SOD/CAT pathways	Requires appropriate catalytic centers and reducing substrates; activity depends on chemical environment	[32,37]
Regenerative redox cycling	Redox-active metal oxides; polydopamine and related redox-active polymers	Reversible electron acceptance/donation and cycling between oxidation states	Multiple ROS and radical species	Allows repeated catalytic antioxidant action rather than one-time stoichiometric scavenging	Sensitive to pH, oxygen, reductants, ligands, surface state and surrounding biomolecules	[33,34,35,36]
Metal-ion regulation	Polydopamine; carbonaceous, polymeric and biomolecule-functionalized nanoparticles	Coordination/chelation of redox-active metal ions	Fe^2+^, Cu^+^ and metal-mediated ROS pathways	Reduces availability of catalytic metal ions and suppresses secondary radical formation	Binding strength and selectivity depend on surface hydroxyl, catechol, carboxyl, amino and related groups	[35,36]
Antioxidant protection and delivery	Polymeric nanoparticles; liposomes/lipid carriers; hybrid nanocarriers	Encapsulation, stabilization, targeted/controlled or stimuli-responsive release	Molecular antioxidants and oxidative-stress microenvironments	Improves antioxidant stability, solubility, localization and duration of action	Release kinetics, carrier stability, loading, targeting and biological compatibility must be optimized	[25,38]
Integrated redox regulation	Multifunctional and multi-enzyme-mimetic nanoantioxidants	Combined radical scavenging, SOD/CAT/GPx-like catalysis, metal regulation and/or delivery	Heterogeneous ROS networks	Provides broader redox control and can better mimic cooperative endogenous antioxidant defenses	Mechanistic multiplicity complicates assay interpretation; net ROS balance and context must be established	[32,33,34,37]

## Data Availability

No new data were created or analyzed in this review. Data sharing is not applicable to this article.

## Abbreviations

The following abbreviations are used in this manuscript:

ROS	Reactive Oxygen Species
RNS	Reactive Nitrogen Species
SOD	Superoxide Dismutase
CAT	Catalase
PEG	Polyethylene Glycol
GPx	Glutathione Peroxidase
PLGA	Poly(lactic-co-glycolic acid)
CeO_2_NPs	Cerium Oxide Nanoparticles
POD	Peroxidase
ET	Electron Transfer
HAT	H-atom Transfer
PDA	Polydopamine
UV	Ultraviolet
Nrf2	Nuclear factor erythroid 2-related factor 2
ARE	Antioxidant Response Element
